# Zinc phthalocyanine loaded- antibody functionalized nanoparticles enhance photodynamic therapy in monolayer (2-D) and multicellular tumour spheroid (3-D) cell cultures

**DOI:** 10.3389/fmolb.2023.1340212

**Published:** 2024-01-08

**Authors:** Nokuphila Winifred Nompumelelo Simelane, Heidi Abrahamse

**Affiliations:** Laser Research Centre, Faculty of Health Sciences, University of Johannesburg, Johannesburg, South Africa

**Keywords:** colorectal cancer, photodynamic therapy, photosensitizers, phthalocyanine, gold nanoparticles, antibody, three-dimensional cell culture models, multicellular spheroids

## Abstract

In conventional photodynamic therapy (PDT), effective delivery of photosensitizers (PS) to cancer cells can be challenging, prompting the exploration of active targeting as a promising strategy to enhance PS delivery. Typically, two-dimensional (2-D) monolayer cell culture models are used for investigating targeted photodynamic therapy. However, despite their ease of use, these cell culture models come with certain limitations due to their structural simplicity when compared to three-dimensional (3-D) cell culture models such as multicellular tumour spheroids (MCTSs). In this study, we prepared gold nanoparticles (AuNPs) that were functionalized with antibodies and loaded with tetra sulphonated zinc phthalocyanine (ZnPcS_4_). Characterization techniques including transmission electron microscopy (TEM) was used to determine the size and morphology of the prepared nanoconjugates. We also conducted a comparative investigation to assess the photodynamic effects of ZnPcS_4_ alone and/or conjugated onto the bioactively functionalized nanodelivery system in colorectal Caco-2 cells cultured in both *in vitro* 2-D monolayers and 3-D MCTSs. TEM micrographs revealed small, well distributed, and spherical shaped nanoparticles. Our results demonstrated that biofunctionalized nanoparticle mediated PDT significantly inhibited cell proliferation and induced apoptosis in Caco-2 cancer monolayers and, to a lesser extent, in Caco-2 MCTSs. Live/dead assays further elucidated the impact of actively targeted nanoparticle-photosensitizer nanoconstruct, revealing enhanced cytotoxicity in 2-D cultures, with a notable increase in dead cells post-PDT. In 3-D spheroids, however, while the presence of targeted nanoparticle-photosensitizer system facilitated improved therapeutic outcomes, the live/dead results showed a higher number of viable cells after PDT treatment compared to their 2-D monolayer counterparts suggesting that MCTSs showed more resistance to PS drug as compared to 2-D monolayers. These findings suggest a high therapeutic potential of the multifunctional nanoparticle as a targeted photosensitizer delivery system in PDT of colorectal cancer. Furthermore, the choice of cell culture model influenced the response of cancer cells to PDT treatment, highlighting the feasibility of using MCTSs for targeted PS delivery to colorectal cancer cells.

## 1 Introduction

Studies have projected that there will be approximately 3.2 million increase in colorectal cancer (CRC) new cases by year 2040, compared to the 1.93 million CRC cases recorded in 2020, worldwide (Xi and Xu, 2021). In just 2020, CRC represented 10% of the total global cancer cases, and among these cases, around 9.4% attributed to cancer related fatalities (Xi and Xu, 2021). CRC pose a significant challenge as the contemporary therapeutic modalities including surgery, chemotherapy and radiotherapy, often yield suboptimal treatment outcomes, particularly when diagnosed at advanced stages with metastases (Xi and Xu, 2021; Gu et al., 2022). Moreover, limitations such as invasiveness, unwanted side effects, lack of specificity and indiscriminately damage to both healthy and cancerous tissues that often emerge reduces initial effectiveness of surgery, chemotherapy, and radiotherapy. This leads to the poor management of CRC and the likehood of resistance (Gu et al., 2022).

Photodynamic therapy (PDT) is a valuable therapeutic intervention for effectively eliminating CRC compared to the aforementioned CRC conventional modalities (Gu et al., 2022). PDT present with notable advantages including selectivity, minimal invasiveness, and reduced adverse effects (Gu et al., 2022; Correia et al., 2021). In PDT, different light-sensitive photosensitizing molecules called photosensitizers (PSs) are usually administered intravenously or topically, and they accumulate within target cells or tissues (Abramczyk et al., 2017; Lima and Reis, 2023). The PSs remain inert in targeted cancerous tissues, however, once they are exposed to specific wavelengths of laser light, they are activated (Lima and Reis, 2023). Upon light activation, photosensitizers respond by undergoing a series of photochemical reactions, thereby generating cytotoxic reactive oxygen species (ROS) in the process (Abramczyk et al., 2017). The ROS then inflict photodamage to the targeted cells, leading to apoptosis (cell death) or enabling the destruction of abnormal tissues (Correia et al., 2021; Zheng et al., 2020). Zinc tetrasulfonated phthalocyanine (ZnPcS_4_) PSs are second-generation photosensitizers that have shown antitumour effects in various cancers in the presence of light activation (Abramczyk et al., 2017; Lima and Reis, 2023). They have received substantial attention due to their attractive chemical and optical characteristics they possess (Abramczyk et al., 2017; Lima and Reis, 2023).

Although PDT has been approved for use in clinical settings, it is still underutilized due to several limitations often related to the inherent characteristic of classical PSs (Gunaydin et al., 2021; Van Straten et al., 2017). These constraints include; prolonged skin photosensitivity, tendency to aggregate under physiological environment and poor tissue penetration of light, all of which impede the general effectiveness of PDT (Winifred Nompumelelo Simelane and Abrahamse, 2021). And so, efforts have been undertaken to improve the selectivity of PSs, their bioavailability, stability and ability to solubilize in physiological conditions through the use of various nanoparticles (NPs). Additionally, the use of NPs as favourable PS delivery platforms has been explored to improve the accumulation of PSs in tumours, PS precise localization, and overall effectiveness. These strategies aim to facilitate the targeted delivery of PSs to the designated site of colorectal cancer tissues (Winifred Nompumelelo Simelane and Abrahamse, 2021). These NP nanocarrier platforms for delivering photosensitizers encompass a numerous range of materials including gold nanoparticles (AuNPs), as reported in previous studies (Winifred Nompumelelo Simelane and Abrahamse, 2021; Li et al., 2022; Sarkis et al., 2023). AuNPs exhibit distinctive physicochemical properties, characterized by their ability to readily form thiol and amine bonds, and their possession of an inherent surface plasmon resonance (SPR) (Li et al., 2022). These distinct characteristics endow them with remarkable versatility that enable for their precise tuning to align with specific wavelengths of visible or infrared light in PDT applications (Li et al., 2022; Hammami et al., 2021). Furthermore, AuNPs exhibit biocompatibility, primarily due to the inert nature of their core, making them relatively non-toxic to cells (Hammami et al., 2021).

The NP-PS delivery approach to improve PS drug delivery in CRC cells by enhancing PS drug accumulation can be accomplished through either passive or active targeting strategy (Winifred Nompumelelo Simelane and Abrahamse, 2021). In all cases, the primary goal is to selectively target cells while minimizing the impact on non-cancerous cells, thereby reducing systemic side effects (Winifred Nompumelelo Simelane and Abrahamse, 2021). The enhanced permeability and retention (EPR) effect which is a result of leaky vasculature, poor lymphatic drainage, and increased vessel permeability, is traditionally recognized as the predominant mechanism for NP progressively accumulation in tumour sites, and thus achieve passive targeting delivery of PSs (Jain et al., 2022). Nevertheless, targeting photosensitizers via EPR effect struggle to solely distinguish between cancerous and healthy neighbouring tissues (Itoo et al., 2022). This leads to somewhat limited target specificity and potential adverse reactions (Zheng et al., 2020).

Active targeting approach capitalizes on specific ligands or moieties modified on the surfaces of NP-PS carriers (Wang et al., 2021). These actively targeting ligands or moieties such as aptamers, carbohydrates, folic acid or antibodies exhibit high selectivity and affinity for specific receptors on targeted cancerous cells, thus improving PSs delivery and minimizes collateral damage to nearby healthy cells (Wang et al., 2021). In several studies, actively targeted NP-PS nanoplatforms have been shown to be more efficient in increasing PS cellular internalization, reduce toxic effects of PS drugs as well as enhance the overall PDT therapeutic efficacy in colorectal cancer cells (Zheng et al., 2020; Winifred Nompumelelo Simelane and Abrahamse, 2021; Montaseri et al., 2022a). Recently, considerable attention has been directed towards functionalizing NP-PS nanoplatforms with unique tumour targeting ligands or moieties such as monoclonal antibodies (mAb) that are meticulously chosen to bind with specific surface molecules or receptors that are overexpressed in cancerous cells, leading to enhanced tumour cells uptake of the PS and PS selectivity (Simelane et al., 2021; Wathoni et al., 2022; Hong et al., 2023). Guanylyl cyclase C (GCC) are transmembrane protein receptors that are expressed on the intestinal epithelial CRC cells, with a high affinity for anti-Guanylate Cyclase (Anti-GCC) mAb, which upon binding can enhance cellular uptake of PSs in CRC (Simelane et al., 2021). These receptors are typically scarce in healthy cells but are significantly present in CRC cells (Simelane et al., 2021; Danaee et al., 2017). The overexpression of Guanylyl cyclase C (GCC) receptors on CRC cancer cells renders them a promising target, that can be efficiently exploited to increase PS internalization in targeted CRC cells, minimize the alteration on nearby healthy cells, thereby enhancing the overall efficacy of PDT (Winifred Nompumelelo Simelane and Abrahamse, 2021).

In the field of PDT therapy, 2-D *in vitro* cell culture models are commonly utilized to investigate mechanisms of action, activity, and properties of PS drugs (Kucinska et al., 2021). Studies have reported on the potential of actively targeted delivery of PS by specific ligands or moieties-functionalized nanoparticles on CRC cells cultured in two-dimensional (2D) systems (Winifred Nompumelelo Simelane and Abrahamse, 2021). Nevertheless, recent findings have indicated several limitations associated with these culture models, such as alterations in cell shape, function, and responses, as well as the absence of interactions between cells and their surroundings (Kucinska et al., 2021; Dalir Abdolahinia and Han, 2023). Consequently, this led to the use of 3-D *in vitro* cell models such as multicellular tumour spheroids (MCTSs) (Mohammad-Hadi et al., 2018). Multicellular tumour spheroids (MCTSs) represent one of the simplest 3-D cell organization models, named after the spherical shape they typically exhibit when cells aggregate without the need for plates or other surfaces to adhere to (Han et al., 2021). These models offer a closer representation to the physiological responses and the actual *in vivo* properties of cells, as well as bridge the gap between *in vitro* experiments and clinical trial outcomes (Chaicharoenaudomrung et al., 2019). Consequently, cells within spheroids exhibit a higher level of functional and morphological differentiation compared to monolayer cells (Rossi and Blasi, 2022).

Therefore, the utilization of MCTSs in CRC PDT treatment is crucial for assessing the photototoxicity potential prompted by PS within actively targeted NP-PS systems allowing for an accurate predictions of their PDT clinical performance (Winifred Nompumelelo Simelane and Abrahamse, 2021). However, there is a deficit in data in the literature on actively targeted PS-NP strategy within *in vitro* cultured MCTSs in CRC PDT therapy (Winifred Nompumelelo Simelane and Abrahamse, 2021). In this study, we offer a comparative analysis of the cytotoxic impacts of a biofunctional nanoconjugates (BNC) composed of ZnPcS_4_ bound to antibody functionalized AuNPs for targeted ZnPcS_4_ PS delivery. We employed anti-Guanylate Cyclase (Anti-GCC) mAb, as specific moieties to achieve active targeting specifically directed and assessed towards both *in vitro* cultured 2-D and 3-D MCTSs cell models derived from Caco-2 colorectal cancer cell line.

## 2 Materials and methods

### 2.1 Materials

Gold (III) chloride trihydrate (HAuCl_4_·3H_2_O, ≥99.9% trace metals basis), tri-sodium citrate (for molecular biology, ≥99%), tannic acid (ACS reagent), SH-PEG2k-NH_2_, Foetal Bovine Serum (FBS), Amphotericin-β, Penicillin-Streptomycin, and Dulbecco’s Modified Eagle’s Medium (D5796) were all acquired from Sigma-Aldrich, Johannesburg, South Africa. TrypLE™ Select Enzyme (1X) (12563-029) ThermoFisher, Johannesburg, South Africa). Hoechst 33258, Caspase 3, and 9 Multiplex Assay kit (Ab219915) as well as 96-well ultra-low attachment plates (174929) were purchased from ThermoFisher, Johannesburg, South Africa. Annexin V/PI apoptosis detection kit (556570) was procured from BD Biosciences, The Scientific Group, Johannesburg, South Africa).

### 2.2 Preparation and characterization of ZnPcS_4_ -antibody functionalized AuNPs

#### 2.2.1 Preparation of ZnPcS_4_ PS

In order to prepare a stock solution, we dissolved 0.0006 g of ZnPcS_4_ powder obtained from SantaCruz^®^ (Biotechnology sc-264509A, Johannesburg, South Africa) in 1 mL of 0.001 M of phosphate-buffered saline (PBS) (Sigma-Aldrich, Johannesburg, South Africa. This resulted in a stock solution with concentration of 0.0005 M. To reach the working concentration of 125 μM, we then diluted this stock solution by adding 4 mL of PBS. After preparation, we covered the solution with foil and stored it at room temperature (Montaseri et al., 2022a).

#### 2.2.2 Synthesis of citrate-AuNPs

1 mL of a 1% AuHCl_4_·3H_2_O solution was introduced into a three-neck flask containing 79 mL of Millipore water in a reflux system. Subsequently, a solution containing 0.5 mL of tannic acid 1%, 4 mL of 1% tri-sodium citrate, and 15.5 mL of Millipore water was added to the flask. The mixture was then agitated at 60°C for a few minutes, resulting in the formation of a red-coloured solution consisting of citrate-coated (AuNPs) (Montaseri et al., 2022b). These synthesized nanoparticles were preserved at a temperature of 4°C for use in subsequent experiments.

#### 2.2.3 PEGylation of AuNPs

To covalently attach SH-PEG-NH_2_ onto the surface of AuNPs, a solution containing 20 mg/mL of PEG in PBS was introduced to 1 mL of citrate-capped AuNPs, initiating a ligand exchange reaction (Montaseri et al., 2022b). This mixture underwent agitation at room temperature for a brief period and was left to incubate for 2 h. Following this, excess SH-PEG-NH_2_ was removed by centrifugation. Subsequently, the AuNPs were reconstituted in PBS.

#### 2.2.4 Conjugation of ZnPcS_4_ to PEGylated AuNPs

1 mL of AuNP-SH-PEG-NH_2_ was combined with 1 mL of 125 µM of ZnPcS_4_ and covered with foil to shield the solution from light exposure. The solution was then gently agitated at room temperature for 24 h using a multifunction vortex mixer (DAIHAN-brand MVM-10) at 1,500 rpm. The day after, the solution underwent robust centrifugation at 15,200 rpm for 1 hour. This high-speed centrifugation effectively caused the AuNPs to bound to the ZnPcS_4_ forming a pellet. Subsequently, the supernatant, which contained any unconjugated ZnPcS_4_, was carefully discarded to eliminate unwanted components. The resulting pellet, composed of ZnPcS_4_ conjugated to AuNP-SH-PEG-NH_2_, was then resuspended in 1 mL of 0.001 M PBS (Phosphate Buffered Saline). Basic characterization techniques were applied to analyze this nanoconjugate. Whenever not in use, this nanoconjugate was stored at a temperature of 4°C.

#### 2.2.5 Attachment of anti GCC to ZnPcS_4_ AuNP-SH-PEG-NH_2_ nanoconjugates

After synthesis of AuNPs and conjugation of ZnPcS_4_, the freely available amine functionalized AuNPs with PEG 2000 of the nanoconjugates were used to attach the cell targeting anti GCC, which was activated via 1-Ethyl-3-(3-dimethylaminopropyl) carbodiimide/N-hydroxy succinimide coupling reaction to form amide bonds between carboxyl groups of Anti GCC and amino groups of the nanoconjugates (Montaseri et al., 2022b). The excess anti GCC mAbs were separated by centrifugation at 15,000 rpm for some minutes and the prepared nanoconjugates were dissolved in PBS to investigate their PDT effect on Caco-2 monolayers and MCTSs cancer cells. The final targeted nanoconjugates were denoted as BNC throughout the study.

#### 2.2.6 Characterization of the nanoconjugate

##### 2.2.6.1 UV-vis spectrophotometry

The spectroscopic characteristics were examined by utilizing a Jenway Genova Nano Plus Life Science Spectrophotometer (Cole-Parmer Ltd. in Stone, Staffordshire, UK). This analysis involved scanning the various components and nanoconjugate within the wavelength range of 300–800 nm at intervals of 1 nm to confirm the presence of distinct absorption peaks at specific wavelengths. All test samples were compared against a 0.001 M PBS blank solution within a 1 mL UV fused quartz cuvette. The resulting spectral data was recorded and subsequently visualized on a line graph for further examination and analysis.

##### 2.2.6.2 Proton (^1^H NMR) and carbon (^13^C NMR) spectra analysis

Proton (^1^H NMR) and carbon (^13^C NMR) Nuclear Magnetic Resonance spectra were recorded at 400 MHz and 100 MHz respectively, using the Agilent Varian NMR spectroscopy (Tshwane University of Technology, Pretoria), and the solvent used was DMSO-d6. The chemical shifts are given in parts per million (ppm) on a delta scale. Data for ^1^H NMR are reported as follows: s = singlet, d = doublet, t = triplet, m = multiplet. Characterization is presented as follows; chemical shift (splitting pattern, coupling, integration).

##### 2.2.6.3 Transmission electron microscopy

The morphology and size of the nanoconjugates were analyzed using a JEM-2100 High Resolution Transmission Electron Microscope (HR-TEM) (manufactured in JEOL Ltd. in Tokyo, Japan). To prepare the samples, they were first sonicated for a duration of 15 min and then carefully deposited onto carbon-coated TEM grids with a mesh size of 200 (Lot# 1261229, provided by SPI Supplies) in a dropwise manner. Subsequently, these grids were allowed to air-dry in a light-protected environment. Once dry, the prepared grids were placed into the microscope, and images were acquired.

### 2.3 *In vitro* cell line models

#### 2.3.1 Caco-2 monolayers

Human colon cancer cells CaCo-2 (Cellonex Cat SS1402 CCAC-FL; CCAC-C) were procured from the American Type Culture Collection. These Caco-2 cells were initially placed in DMEM medium enriched with 10% fetal bovine serum (FBS), a solution of 100 U of penicillin and 100 μg/mL of streptomycin, 2.5 μg/mL of amphotericin B and 1 mM sodium pyruvate. The monolayer cell culture models were subsequently incubated at 37°C in an environment consisting of 5% CO_2_ and 85% humidified air. After incubation, the cells were seeded at a density of 6 × 10^5^ cells per 3 mL in 35mm- diameter culture dishes and allowed to attach for 24 h before initiating *in vitro* cellular experiments for photodynamic therapy.

#### 2.3.2 Multicellular tumour spheroid culture (MCTSs)

Human colorectal cancer cells (Caco-2), obtained from Cellonex were cultivated in DMEM media enriched with 10% Fetal Bovine Serum (FBS) and 0.1% of both penicillin-streptomycin and amphotericin-β and 1 mM sodium pyruvate. The Caco-2 cells were cultured in a T-75 flask and maintained at 37°C, with 5% CO_2_ conditions. Once they reached approximately 80% confluence, the cells were collected from the T-75 flask and subsequently seeded into a 96-well - ultra-low attachment plate. In each well, 5,000 cells were plated in 200 μL of medium. The cells were then incubated for a period ranging from 3-4 days, allowing the multicellular tumor spheroids (MCTSs) to grow until they reached an average diameter of 500 μm.

### 2.4 Photodynamic cellular experiments

#### 2.4.1 Morphology

Within the study, we used a Wirsan Olympus CKX 41 inverted light microscope to examine the morphological characteristics of Caco-2 monolayers and MCTSs. Images were taken with a digital camera attached to the Wirsam Olympus CKX41 microscope (Johannesburg, South Africa).

#### 2.4.2 PDT *in vitro*

Monolayers and MCTSs were incubated with equivalent IC_50_ concentrations of 0.125 µM or 3 µM ZnPcS_4_, ZnPcS_4_-AuNPs or ZnPcS_4_-AuNPs-anti GCC (BNC) diluted in cell media achieving a total volume of 3 mL or 200 µL respectively (depending on each experiment). The cells or MCTSs were incubated for 24 h and after this time the monolayers and MCTSs were washed with PBS to remove the free BNC and the medium refreshed. The cells or MCTSs were exposed to irradiation using a semiconductor diode laser (provided and installed by the Council for Scientific and Industrial Research (CSIR)-National Laser Centre (NLC), South Africa) at a light energy dose of 10 J/cm^2^ (irradiance: 9.5 mW/cm^2^, 17 min) with a wavelength of 673 nm.

#### 2.4.3 Adenosine triphosphate (ATP)

The CellTiter-Glo™ 3D luminescence kit (Promega, G968A, Madison, WI, United States) Kit was employed to assess the intracellular ATP levels within the monolayers and MCTSs. MCTSs were transferred into microcentrifuge tubes and disassembled by exposing them to 200 μL of TE solution at 37°C for a duration of 30 min while maintaining continuous agitation. Subsequently, 200 μL of HBSS was introduced to stop the reaction. The tubes were then subjected to centrifugation at 2,500 rpm for 5 min, and the supernatant was discarded. Following this, the cells were re-suspended in HBSS. 100 μL of the cell suspension was transferred to an opaque 96-well plate, with an equal volume of ATP substrate was also added. The contents were gently agitated for 5 min to enhance reagent penetration and cell lysis. The samples were subsequently incubated at room temperature for an additional 25 min, and the luminescence emanating from intracellular ATP was measured using the Victor Nivo^®^ multimode plate reader (Perkin-Elmer, Midrand, South Africa).

#### 2.4.4 Cell death

To ascertain the mode of cell death, 24 h post-treatment with either the PS, ZnPcs4-AuNPs or BNC nanoconjugates in the absence of light or following laser exposure, the monolayers and MCTSs were dispersed using TE. Subsequently, single-cell suspensions from both the control and experimental groups underwent centrifugation, leading to the removal of supernatants. The cells were then subjected to 2 times rinse with PBS before being suspended in 1× binding buffer, and then kept at low temperatures.100 μL of the cell suspension was transferred to a flow cytometry tube and incubated in the absence of light with 5 µL of Annexin V-FITC and 5 µL of propidium iodide stains. After gentle vortexing and a 15-min incubation at room temperature, the cell preparations were examined using a Becton Dickinson (BD) Accuri C6 flow cytometer, with each tube receiving the addition of 400 µL of 1× binding buffer.

#### 2.4.5 Fluorometric quantification of Caspase-3, and 9 activities

Cell suspensions from various control and experimental groups that were obtained 24 h after PDT treatment were mixed with caspase solution in a poly-D-lysine-coated plate, followed by 1 h of incubation at room temperature in the dark. The PerkinElmer VICTOR Nivo™ was used to measure the fluorescence at specific wavelengths: Ex/Em = 535/620 nm (Caspase 3) and Ex/Em = 370/450 nm (Caspase 9). The results for each experimental group were reported as a fold increase in caspase levels versus the untreated control.

#### 2.4.6 Live/dead assay

The control and experimental groups of monolayers and MCTSs were subjected to three washes with PBS and subsequently underwent staining with a solution containing 1 μg/mL of ethidium bromide (EtBr) and an equivalent concentration of acridine orange (AO) for a duration of 5 min, all in PBS. Following this staining step, the monolayers and MCTSs were rinsed three times with PBS and observed using a Carl Zeiss fluorescent microscope equipped with Alexa fluor 488 and EtBr channels. The visualization process was facilitated using the Zen Pro (3.7) Carl Zeiss software.

#### 2.4.7 Nuclear damage

Cells were cultured in sterilized coverslips placed in culture dishes. Post treatment, Caco-2 cells were fixed with 4% paraformaldehyde then stained with Hoechst 33258 (1 μg/mL) in 1 mL of culture media for 5 min at 21°C (room temperature). The coverslips were rinsed three times with PBS, mounted on glass slides, and a fluorescence microscopy was used to capture images.

### 2.5 Statistical analysis

The differences between the control and experimental groups underwent one-way analysis of variance (ANOVA) followed by the Dunnett test using Sigma Plot version 12. All the results are expressed as the mean ± standard error obtained from three separate experiments. Statistically significance was determined, wherein * represents *p* < 0.05, ** indicates *p* < 0.01, and ***p < 0.001.

## 3 Results

### 3.1 Characterization of the biofunctional nanobioconjugate

#### 3.1.1 UV-visible spectroscopy

##### 3.1.1.1 ZnPcS_4_ PS

**FIGURE 1:**
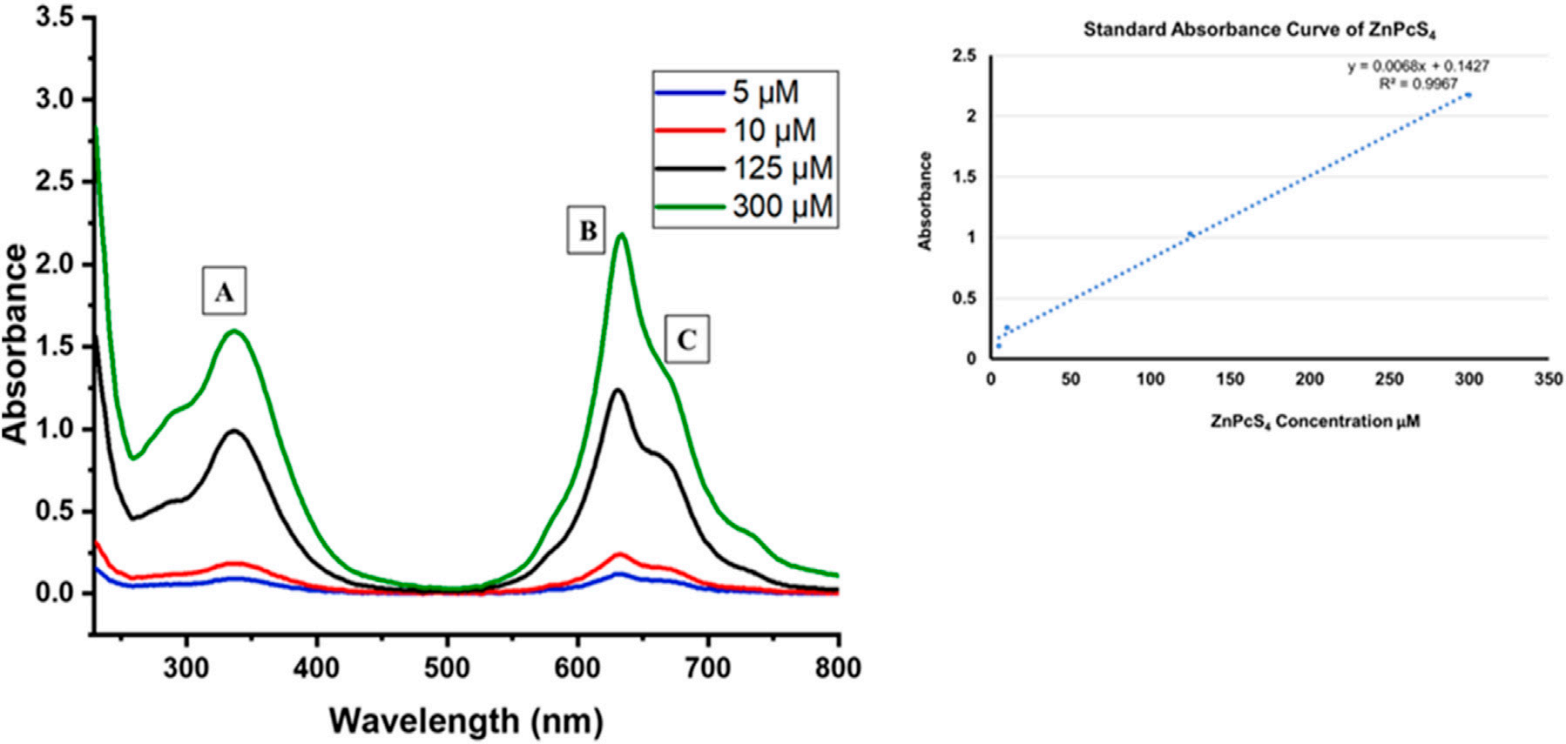
The UV–visible absorption spectra of unbound ZnPcS4 in PBS aqueous solution, at various concentrations of PS.

Figure 1 displays the UV–visible absorption spectra of ZnPcS_4_ at different concentrations. Notably, distinct absorption peaks within the Q band are evident around 634 nm and 673 nm (Figures 1B, C), while the B band exhibits an absorption peak at approximately 336 nm (Figure 1A). These UV-Vis peaks observed in PBS are characteristic of the signature peaks associated with ZnPcS_4_ PS. Furthermore, ZnPcS_4_ demonstrated well-defined absorption peaks in both the Soret band and Q band, indicative of its excellent solubility in PBS and minimal aggregation. It is noteworthy that these absorption peaks in the Soret band and Q band are typically suited for photodynamic diagnosis (PDD) and photodynamic therapy (PDT) applications, respectively (Simelane et al., 2021; Olszowy et al., 2023). A standard calibration curve was also generated using the absorbance measurements acquired at 673 nm for various concentrations of ZnPcS_4_ PS. A linear regression model was employed to fit the data (y = 0.0068x +0.1427, R2 = 0.9967). Consequently, the calculated concentration of ZnPcS_4_ was determined to be 88.9 µM.

##### 3.1.1.2 PeGylated-AuNPs

**FIGURE 2:**
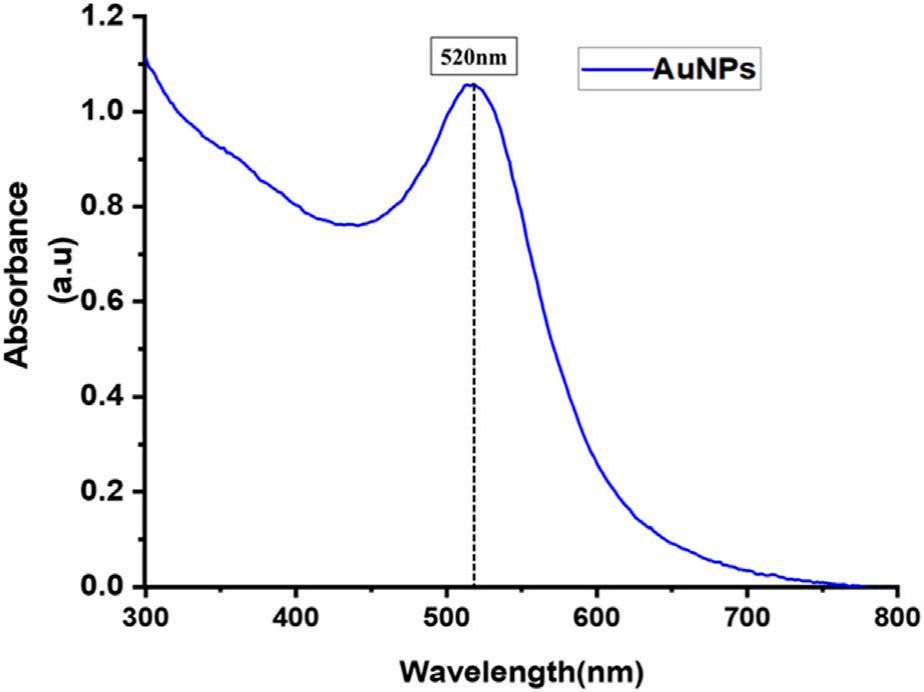
UV-visible spectrum of AuNPs.

The UV-Vis spectrum depicted in Figure 2 clearly displays the typical characteristic peak of PeGylated-AuNPs near 520 nm, which is within the well-recognized characteristic absorption band associated with the surface plasmon resonance peak for spherical gold nanoparticles (Yu et al., 2021). This peak corresponds to the typical range found in the UV Vis spectrum of small, spherical gold nanoparticles.

##### 3.1.1.3 UV-visible spectral analysis of active biofunctional targeted nanoconjugate

**FIGURE 3:**
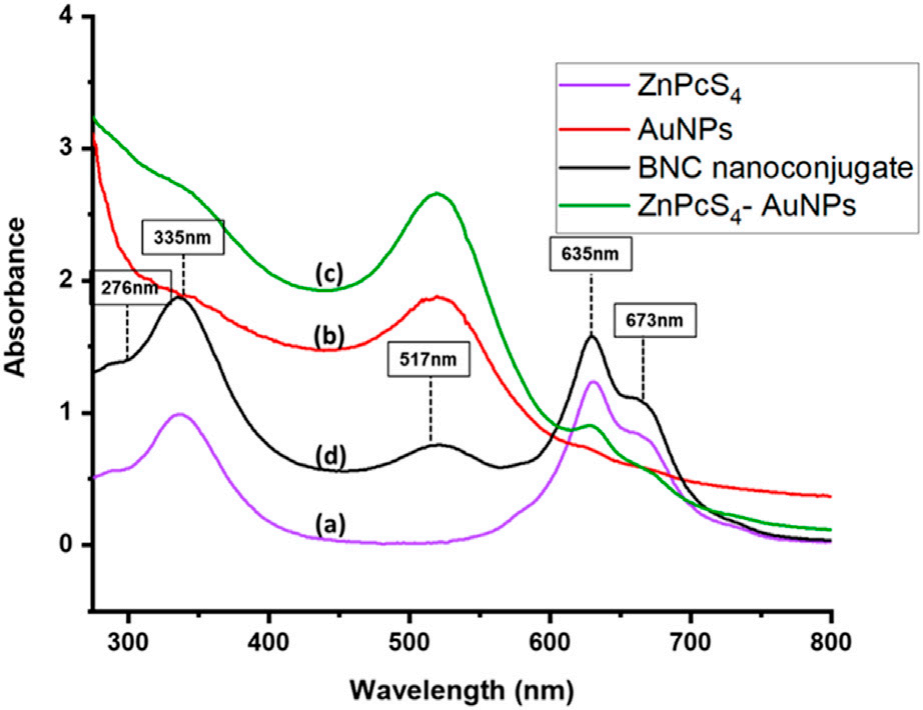
The UV–Vis spectra of (a) ZnPcS_4_ (b) AuNPs (c) ZnPcS_4_-AuNPs and (d) biosynthesized BNC nanoconjugates display distinctive peaks at 335 nm, 635 and 673 nm for ZnPcS_4_ PS, at 517 nm for AuNPs and at 276 nm for anti GCC Ab, in the range from 300 to 850 nm.

The UV–Vis absorption spectra of ZnPcS_4_ (PS), AuNP, anti-GCC Ab, ZnPcS_4_-AuNP, and ZnPcS_4_-AuNP-GCC Ab (BNC) were next recorded in the wavelength range of 200–800 nm using a UV spectrophotometer to study their optical properties, as presented in Figure 3. In Figure 3 (spectrum a), we observed the conventional absorption bands of ZnPcS_4_, with the B band appearing at 340 nm and Q bands at 634 and 673 nm. Figure 3 (spectrum b) illustrates the characteristic absorption peak around 520 nm for the biosynthesized AuNPs. Proteins, including Abs, often exhibit absorption maxima typically in the range of 255–280 nm due to the presence of aromatic amino acids (Prasad et al., 2017). In Figure 3 (spectrum c), the UV-Vis spectral absorbance of anti-GCC Ab is presented, revealing a distinctive peak at 260 nm in the absorption spectrum. The absorption spectra of the bare constituents were subsequently compared with those of ZnPcS_4_-AuNP and the biofunctional multicomponent nanobioconjugate, ZnPcS_4_-AuNP-Ab (BNC). The absorption peaks of BNC closely resembled those of bare ZnPcS_4_, AuNP, and PS-AuNP, exhibiting the characteristic features of both ZnPcS_4_ and AuNPs. However, the absorption peak of the PS within the PS-AuNPs exhibited a distinct broadening, with a Soret band peaking at 335 nm and Q bands at 635 nm and 673 nm following the AuNP attachment. This shift can be ascribed to an increase in nanoparticle size, indicating the successful binding of ZnPcS_4_ PS to AuNPs. In the BNC nanoconjugates, the observed absorption peaks remained consistent with those found in the spectrum of ZnPcS_4_ PS. The ZnPcS_4_ in the nanoconjugates displayed broader peaks around 335 nm, 635 nm, and 673 nm, suggesting that the PS retained its inherent PDD and PDT capabilities even after conjugation (Simelane et al., 2021).Within the UV/Vis absorption spectrum of the BNC conjugate, clear evidence of the presence of AuNPs and anti-GCC Ab was observed. The resonance peak attributed to AuNPs exhibited broadening, with decreasing absorption peak at 517 nm. A pronounced peak at around 276 nm, indicative of the presence of anti-GCC Ab within the BNC nanoconjugates’ spectrum was also observed. These findings suggest that all three individual constituents were successfully linked (Simelane et al., 2021).

#### 3.1.2 Proton (^1^H NMR) and carbon (^13^C NMR) spectra analysis

The Nuclear Magnetic Resonance spectra for Proton (^1^H NMR) and carbon (^13^C NMR) were acquired at 400 MHz and 100 MHz, respectively, and the solvent used was DMSO-d_6_. Chemical shifts were expressed in parts per million (ppm) on a delta scale (δ). Data for ^1^H NMR was reported as follows: s = singlet, d = doublet, t = triplet, m = multiplet and characterization was presented as follows; chemical shift (splitting pattern, coupling, integration). Within the PEG-Thio-Amine (Standard), the ^1^H NMR spectrum (Supplementary Figure S1) displayed a triplet observed upfield at 2.96 ppm which is indicative of the hydrogens attached to the S-H fragment. This splitting pattern caused by the adjacent protons is particularly significant as it is directly attached to the sulfur atom of the S-H end of the moiety. Additionally, a singlet was observed at 3.32 ppm and a multiplet appeared at 3.46–3.52 ppm, which corresponded to the protons on alkoxy carbons of the repeating units of the molecule. The carbon attached to S-H was also observed upfield at 21 ppm in the ^13^C NMR spectrum (Supplementary Figure S2) and alkoxy carbons of the repeating units were observed as a singlet, which appeared at 70.23 ppm.

**FIGURE 4:**
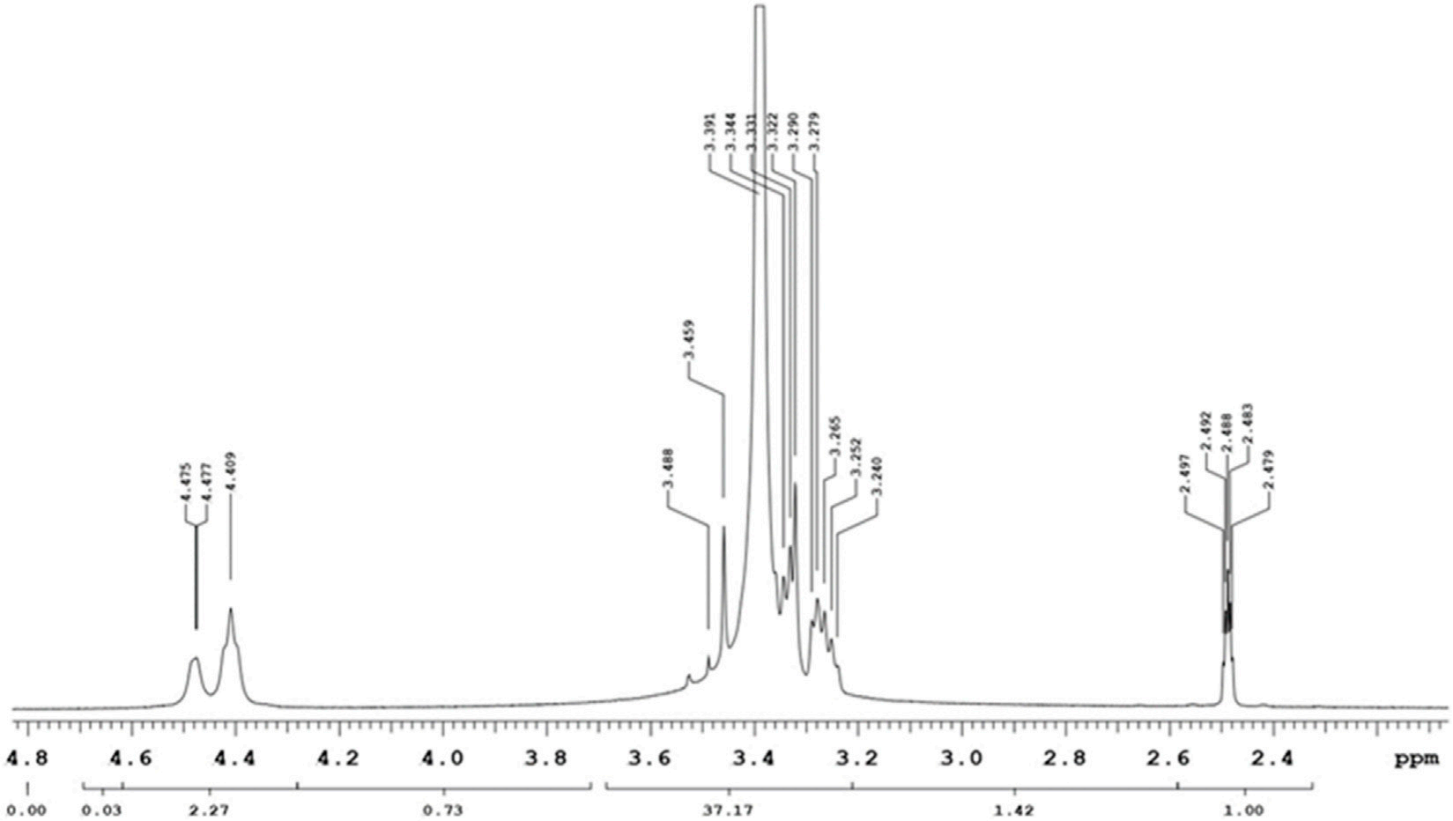
^1^H-NMR of -PEG-AuNPs-anti GCC.

**FIGURE 5:**
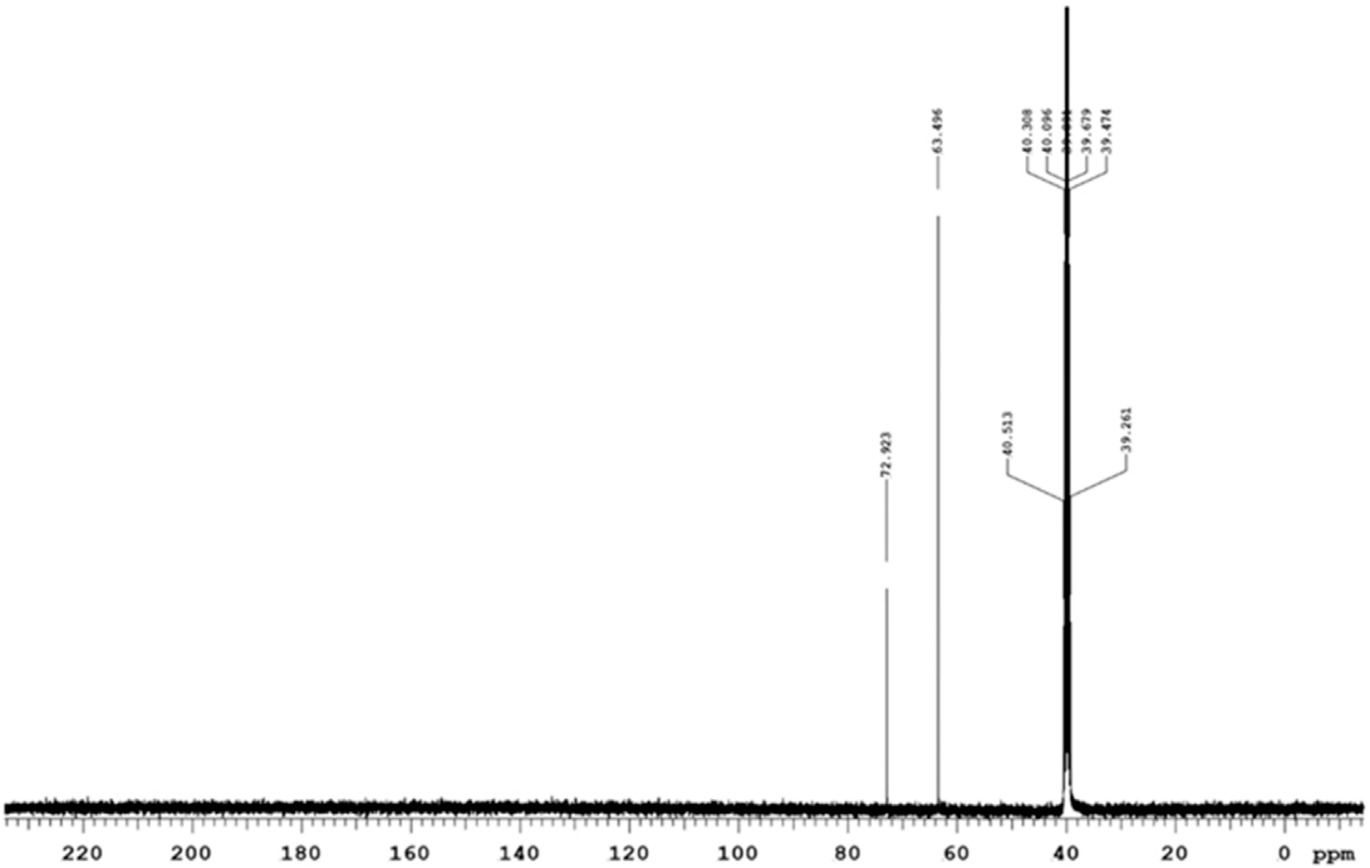
^13^C NMR spectrum of PEG-AuNPs-Anti GCC.

The ^1^H NMR spectrum of the PEG-AuNP-Antibody complex depicted in Figure 4, showed evidence of conjugation (coordination) between AuNP and the PEG-thio-amine and antibody. This was seen as the absence of the triplet typically observed in the ^1^H NMR spectra for PEG-Thio-amine. Furthermore, broadening of the multiplets in the range of 3.24–3.48, along with a slight shift (0.22 ppm) towards the upfiled was observed for the protons on the alkoxy carbons within the repeating units of the molecule. In the ^13^C NMR spectrum (Figure 5), there were no peaks observed due to C-S bond between 20-21 ppm. The change in chemical shift and the broadening of the peaks can be attributed to the proximity of the nanoparticle surface in which it forms Au-S bond with gold thereby eliminating the sulfide hydrogen. Likewise, on the protons of the alkoxy carbons, a chemical shift upfield at 63.49 ppm was observed for the alkoxy carbons of the PEG-thio-amine molecule attached to the AuNP. Additionally, an extra carbon peak was discernible at 72.93 ppm in the ^13^C NMR indicative of the presence of an extra molecule (antibody) in the complex.

#### 3.1.3 TEM

**FIGURE 6:**
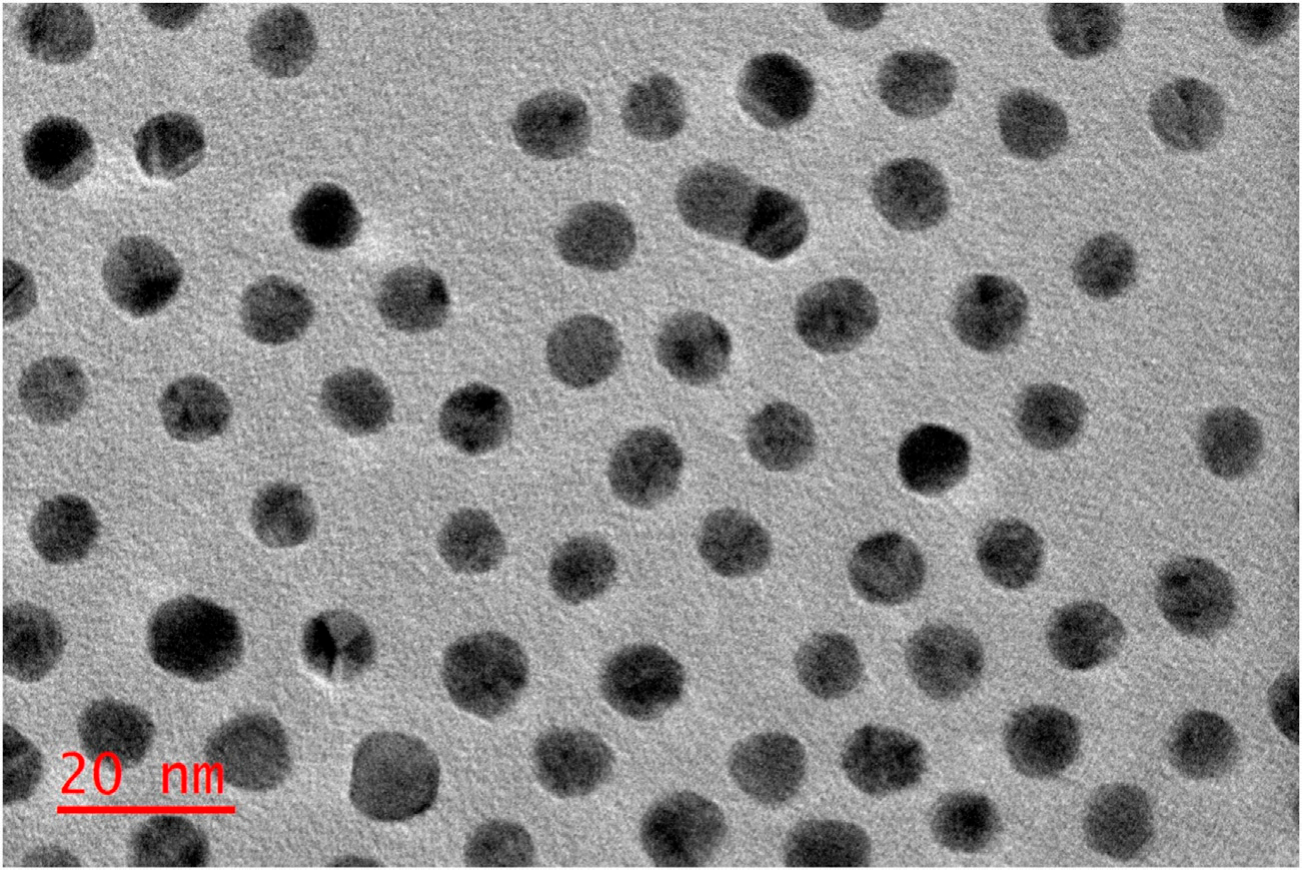
Images obtained from Transmission Electron Microscopy, representing the ZnPcS_4_- GCC- loaded AuNPs (BNC) compound.

The nanoconjugates shape and size distribution were investigated using the HRTEM. As depicted in Figure 6, the NPs exhibited spherical morphology shapes. Furthermore, the nanoconjugates exhibited a narrow-small sized distribution, had diameters typically around 13 nm, which is possibly attributed to the loading of the ZnPcS_4_ PS and the Anti GCC Ab decorated on the surface of the spherical PEGylated AuNPs.

### 3.2 Photodynamic effects of the BNC *on in vitro* cultured cells

#### 3.2.1 Development of Caco-2 monolayers and MCTSs

**FIGURE 7:**
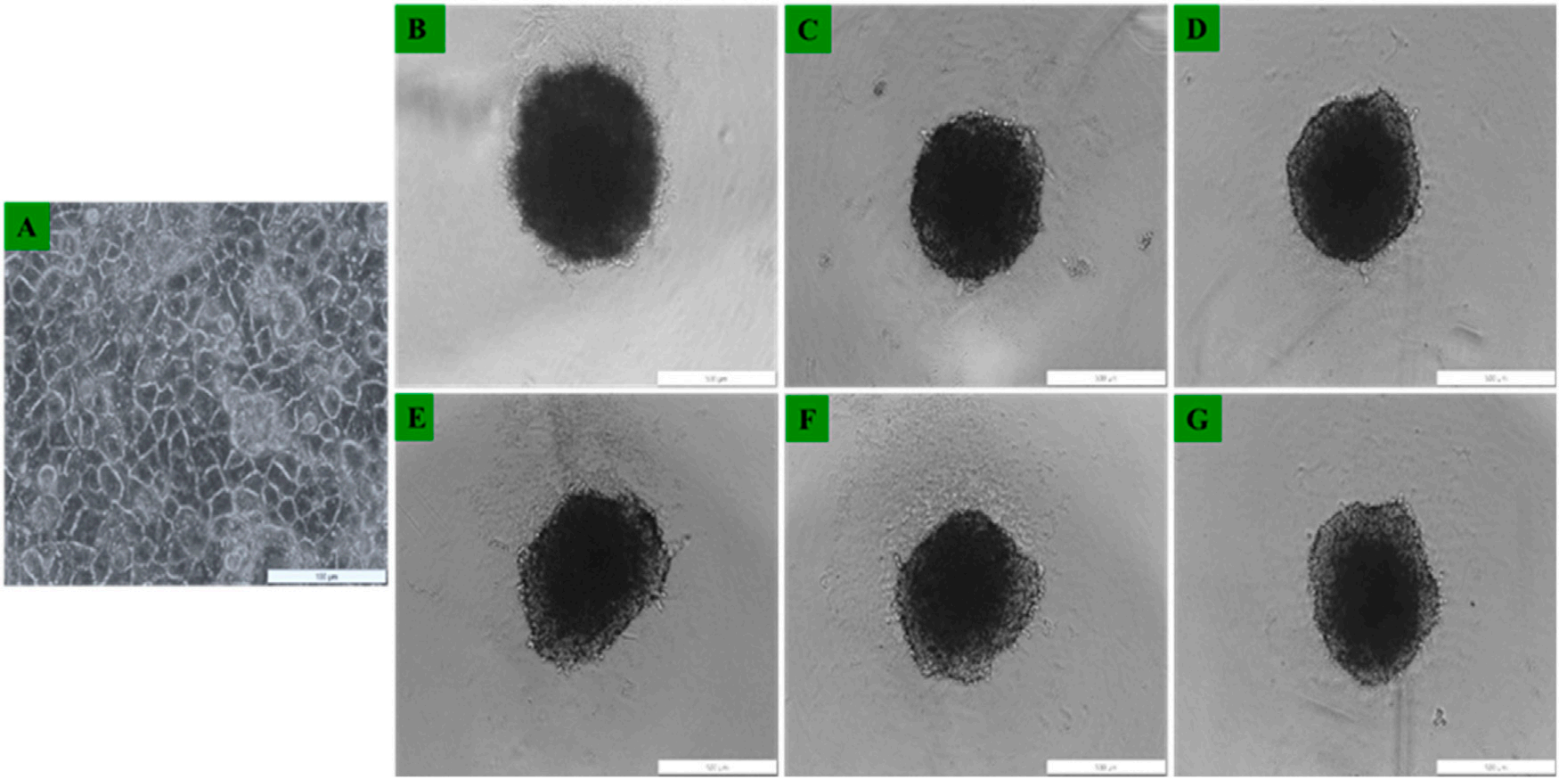
Representative images of Caco-2 cells morphological assessment of **(A)** Caco-2 grown as 2D monolayers 24 h post seeding, and Caco-2 MCTSs at **(B–D)** day 3 and at **(E–G)** day 5) seen under ×4 objective.

Morphological evaluation of Caco-2 monolayers and MCTSs cultured in culture dishes and 96 well low attachment plates was performed using the light inverted microscopy. Caco-2 monolayers adhered to the culture plate surfaces within 24 h post seeding. Morphological examination through the inverted light microscopy revealed no discernible damage, with the cells retaining their distinctive morphological appearance. Notably, Caco-2 cells had been successfully cultured, observed as single cell layers in monolayers (Figure 7A), and as Caco-2 MCTSs which were successfully developed using the low attachment 96 well plates -overlay technique (Figures 7B–G). As can be seen in Figures 7B–G, MCTSs exhibited compactness from day 3 and day 5. The spheroids exhibited varying shapes, and their average size ranged between 300 and 500 µm. There were no significant differences observed in terms of their sizes, in Day 3 and day 5 -MCTSs.

#### 3.2.2 Adenosine triphosphate (ATP) assay in Caco-2 monolayers and MCTSs

Cell proliferation and DNA replication rely on the mitochondrial metabolism to generate ATP as an energy source (Xiang et al., 2021). In order to study the changes in ATP levels of ZnPcS_4_-AuNPs and BNC nanoconjugates in monolayers and MCTSs with or without 673 nm light treatment after incubation with or without equivalent concentrations of 0.125 µM ZnPcS_4_ in monolayers or 3 µM ZnPcS_4_ in MCTSs, the adenosine triphosphate (ATP) Cell Titer-Glo^®^ luminescent assay was used to assess the ATP content level and impact of PDT on cell lines when cultured as monolayers (2D) or as MCTSs (3D).

**FIGURE 8:**
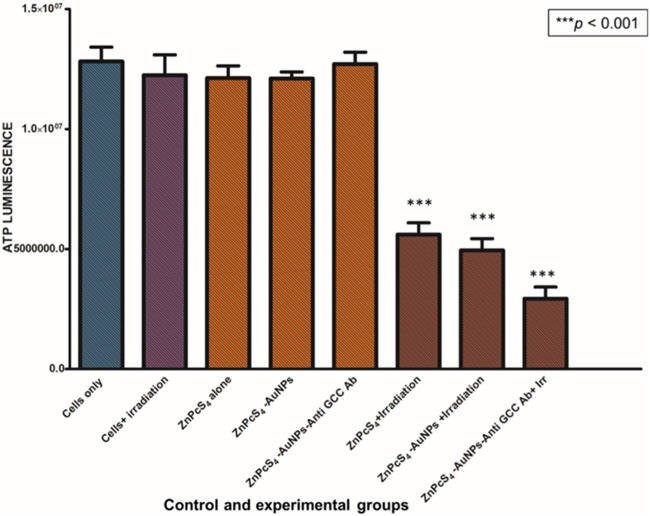
ATP level evaluation following PDT after incubation with ZnPcS_4,_ ZnPcS_4_-AuNP and BNC. Untreated Caco-2 cells were used as control. The results are expressed as the average values ±SEM (*n* ≥ 3). The statistical analyses were performed through the One-way Anova and Dunnett method (***p < 0.001).

**FIGURE 9:**
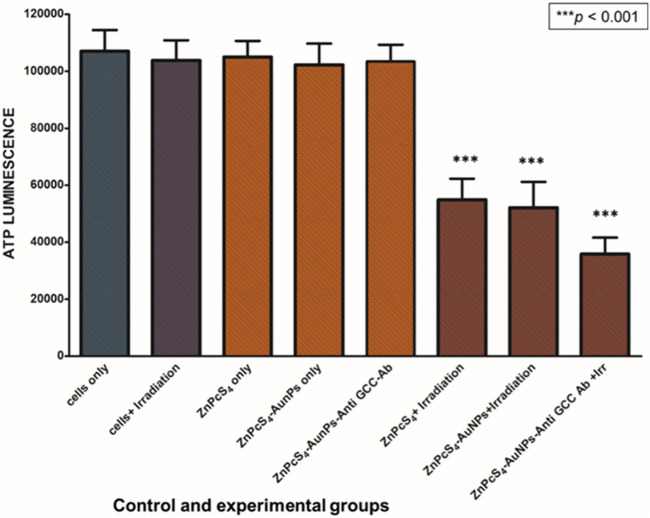
Cellular ATP levels of Caco-2 MCTSs treated with or without ZnPcS_4_-AuNP-Anti-GCC and at 24 h after laser irradiation.

As depicted in Figures 8, 9, Caco-2 monolayers or Caco-2 MCTSs control groups incubated with free ZnPcS_4_, nanoconjugates alone or those exposed solely to laser irradiation alone, displaying higher ATP levels when compared to treated cells, suggesting that these treatment conditions did not affect the proliferative activity of monolayers or spheroids cultured cancer cells (Figures 8, 9). Conversely, PDT-mediated treatment led to a reduction of ATP content levels, in 2D monolayers treated cells or MCTSs compared to that in the untreated control groups. Specifically, ZnPcS_4_-AuNP and BNC treated groups resulted in a significant decrease in intracellular ATP levels (***p < 0.001). These results indicate that ZnPcS_4_-AuNP and BNC possess a stronger antiproliferative activity in Caco-2 cell lines owing to their targeting abilities facilitated by nanoparticles and targeting moieties. ATP levels were more marked in monolayers compared to MCTSs. In addition, according to the IC_50_ values, the IC _50_ of ZnPcS_4_ for monolayers was lower than MCTSs, thus were considerably more prone to cell death and demonstrated higher sensitivity to the BNC nanoconjugates than their counterparts in MCTSs cultures, while under the same experimental conditions of 673 nm light exposure, MCTSs were less susceptible to BNC (IC_50_ = 3 µM) than their counterparts cultured in 2D models (IC_50_ = 0.125 µM). The cell viabilities decreased with the increase of equivalent ZnPcS_4_ concentration (3 µM) in MCTSs, indicating that the spheroids were more resistant to lower dosages that were typically effective in monolayers.

#### 3.2.3 Cell death mechanism analysis using flow cytometry in Caco-2 monolayers and MCTSs

**FIGURE 10:**
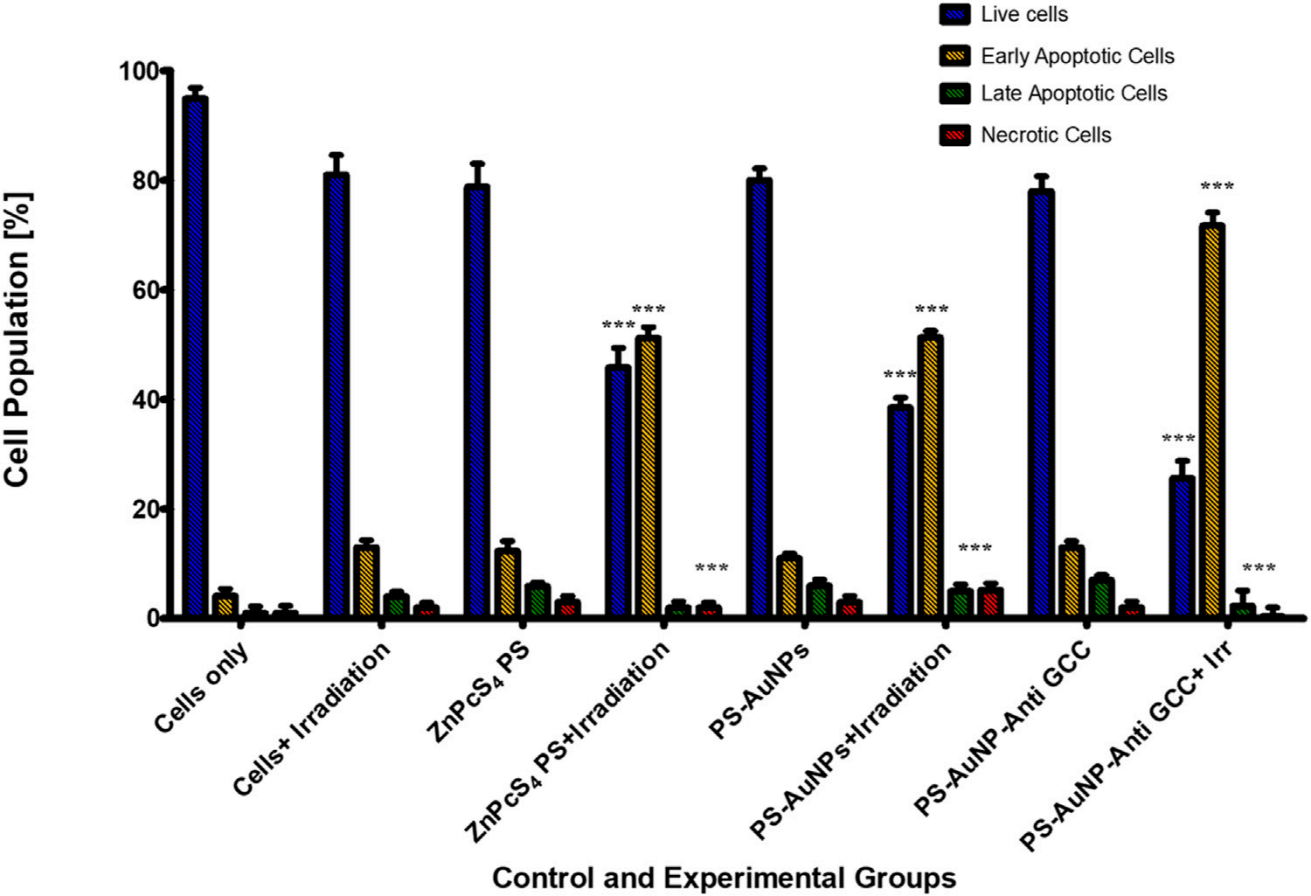
Cell apoptosis measured by flow cytometry using annexin V-FITC and PI double staining. Caco-2 cells were exposed to 0.125 μM of either ZnPcS4, ZnPcS4-AuNPs, or BNC and laser irradiation. Untreated Caco-2 cells and cells exposed to either free ZnPcS4, ZnPcS4-AuNPs alone, or BNC alone were used as control. The statistical analyses were performed through the One-way Anova and Dunnett method (***p < 0.001).

To determine the mode of cell death mechanism elicited, which is related to the cytotoxic effect of ZnPcS_4_, ZnPcS_4_-AuNPs, or BNC nanoconjugates exposed to laser irradiation treated Caco-2 monolayer cells and MCTSs were analyzed by flow cytometry, and Annexin-V and PI double staining techniques was utilized to confirm the cell death mechanism induced (Figure 10). As shown in Figure 10, within untreated control groups, the population of Caco-2 cells residing in the apoptotic quadrant was extremely low, untreated cells exhibited high cellular viability (94.9%). Similarly, more than 60% of cells remained viable following incubation for 24 h with ZnPcS_4_, ZnPcS_4_ -AuNPs, or BNC nanoconjugates alone. However, the results show that the cell apoptotic rates of Caco-2 cells in the experimental groups treated with either ZnPcS_4_, ZnPcS_4_-AuNPs, or BNC nanoconjugates in the presence of laser irradiation were significantly increased though markedly different. Among the treated groups, the most prominent apoptotic effect on Caco-2 cell lines was observed in BNC nanoconjugates and laser irradiation. BNC treated with laser irradiation showed early apoptosis (71.7%), late apoptosis (2.3%) and necrosis (0.4%). These results indicated that BNC nanoconjugates and laser irradiation could induce and promote cell apoptosis of Caco-2 cells.

**FIGURE 11:**
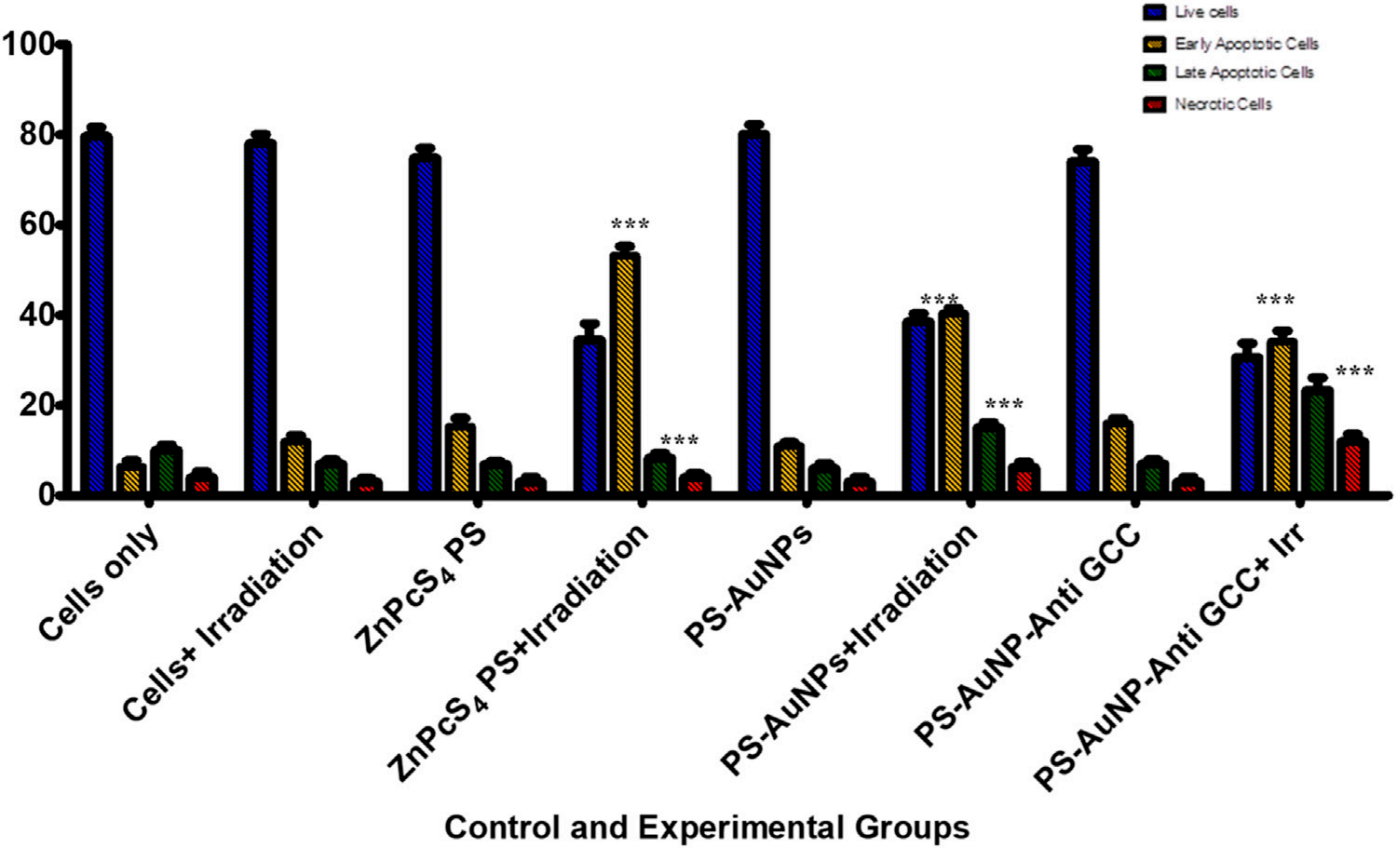
Flow cytometric analysis of apoptosis rate of Caco-2 MCTSs after treatment with either ZnPcS_4_, ZnPcS_4_-AuNPs, or BNC nanoconjugates in the presence of laser light.

To elucidate whether the reduced proliferation of MCTSs would be due to cytotoxic effects of PDT treatment, we then evaluated whether PDT mediated treatment is able to induce apoptosis within MCTSs (Figure 11). The induction of apoptosis was analysed by flow cytometry. Spheroids were exposed to either (3 μM) ZnPcS_4_, ZnPcS_4_-AuNPs, or BNC nanoconjugates in the presence of illumination laser, and 24 h post irradiation, dissociated and incubated with an Annexin-V-FITC and propidium iodide (PI) double stain. ZnPcS_4_ PS-mediated PDT treatment induced a high degree of early (53%) and late apoptosis (8%) respectively, in comparison to untreated MCTSs controls. PDT treatment induced a relatively slight increase in apoptotic response in Caco-2 spheroids, with a slightly stronger effect on Caco-2 MCTSs treated with BNC, where cell death levels showed that about 60% of Caco-2 cells cultured as MCTSs were in apoptotic stages as opposed to 5.4% of untreated cells. In contrast, untreated MCTSs did not display apoptosis. Similarly, MCTSs were not affected by treatment with ZnPcS_4_, ZnPcS_4_-AuNPs, or BNC nanoconjugates alone. As Figure 11 shows, there were no noticeable changes in the number of apoptotic cells in BNC plus absence of light group, (16% of cell death), almost similar to the untreated control. Taking these findings together, Caco-2 2-D monolayer cultures were more prone to cell death induced by BNC mediated PDT than their MCTSs counterparts. Differences in cell susceptibility upon laser light exposure within targeted PDT treatment have also been reported for HCT-116 colon cancer cells (Gibot et al., 2014; Pereira et al., 2017). Several studies have demonstrated that spheroids have a higher resistance to PDT than monolayers (Pereira et al., 2017; Zuchowska et al., 2017). Our results were also consistent with the reduction of ATP levels of treated groups discussed above. Thus, as observed in our study, the susceptibility of Caco-2 cells to the phototoxic effects of BNC -PDT treatment was confirmed in both monolayers and MCTSs, which resulted in cell death by early and late apoptosis.

#### 3.2.4 Measurement of Caspase-3 and Caspase-9 activity in Caco-2 monolayers and MCTSs

**FIGURE 12:**
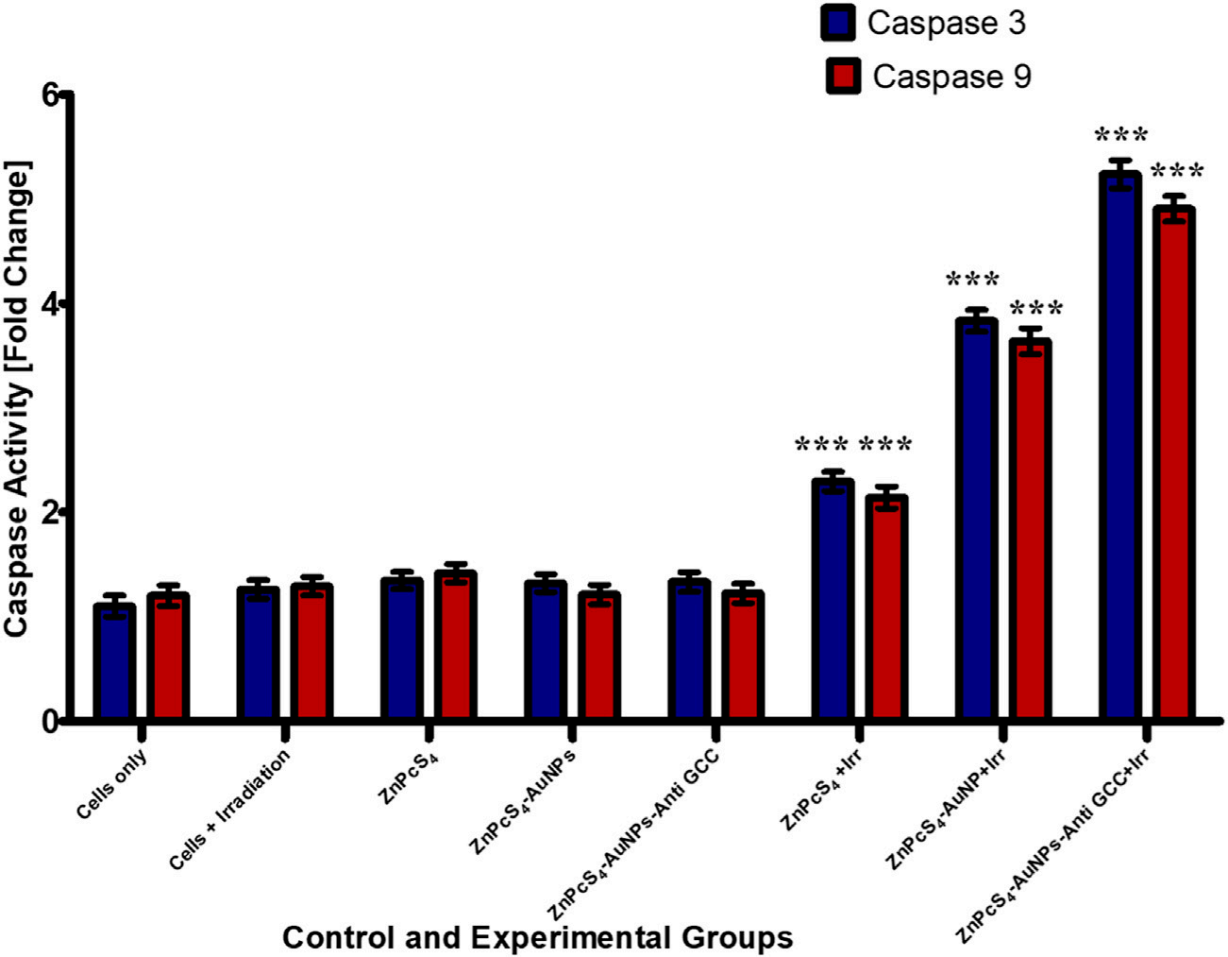
Caspase-3 and caspase-9 activity levels in Caco-2 monolayer cancer cell lines measured 24 h post treated with or without treatment of either ZnPcS_4_, ZnPcS_4_-AuNPs, and/or BNC nanoconjugates + laser irradiation.

Caspases are cysteine proteases that are best known for their ability to play a crucial role in triggering and executing apoptosis (Salmerón et al., 2018). To determine if the targeted PDT-induced apoptosis observed in Caco-2 cells, indeed contributed to Caco-2 cells growth inhibition, the level of the caspases 3 and 9 activities was measured using fluorometric caspase assay in PDT treated Caco-2 cells compared to untreated control population (Figure 12). As observed in Figure 12, caspase 3 and 9 levels were four-fold higher in monolayer cell groups exposed to PDT than those of untreated control group. Moreover, within PDT treated group, caspase-3 and caspase-9 levels were markedly increased by BNC nanoconjugate + laser irradiated-mediated treatment indicating the possible role of BNC nanoconjugate in inducing apoptosis in Caco-2 cells.

**FIGURE 13:**
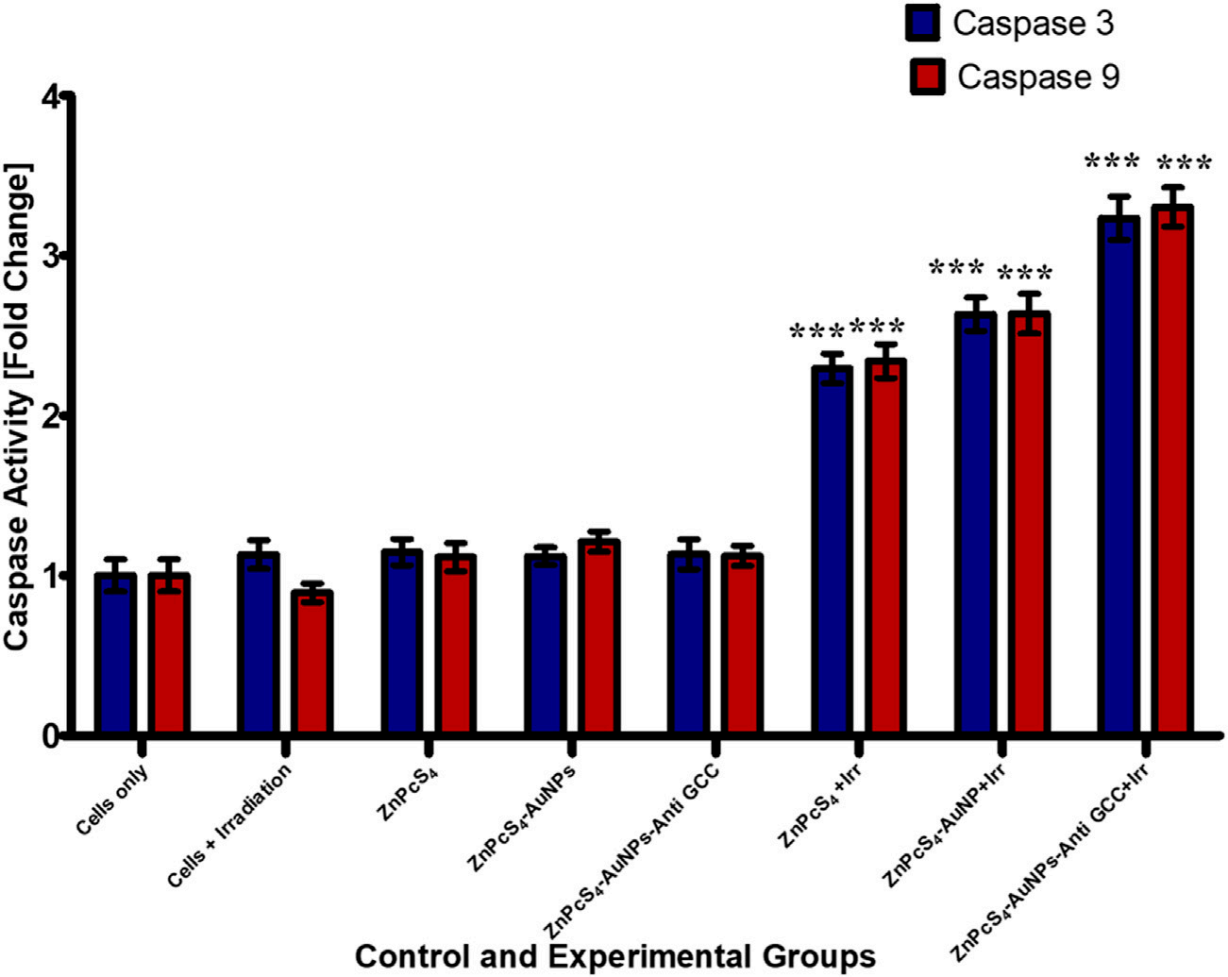
Caspase-3 and caspase −9 activity in Caco-2 MCTSs was determined by fluorometric caspase assay, following a 24 h treatment with ZnPcS_4_, ZnPcS_4_-AuNPs, or BNC nanoconjugates and laser irradiation. Values are means ± SE (*n* = 3), representative of three experiments.

PDT treatment markedly (***p < 0.001) increased caspases 3 and caspase-9 activity levels in all treated Caco-2 cells from 3-D MCTSs relative to the untreated group (Figure 13). MCTSs treated with BNC nanoconjugates plus laser irradiation displayed an evident increase in the activity of these caspases, indicating the involvement of caspases in the initiation of apoptosis. In contrast, no significant effect on caspases 3 and caspase-9 was observed in MCTSs exposure to either free ZnPcS_4_, ZnPcS_4_-AuNPs alone, or BNC nanoconjugate alone, showing the same behaviour as Caco-2 MCTSs of negative untreated spheroid control group. The level of the caspases 3 and 9 activities in Caco-2 monolayers incubated with BNC-PDT was higher than that of MCTSs incubated with BNC which was consistent with the results of our ATP assay and cell death analysis, suggesting that BNC mediated PDT treatment induced more oxidative stress in Caco-2 monolayers than in MCTSs, albeit less effectively. MCTSs from human melanoma A375 cells have also been reported to produced higher levels of caspases 3 and 9 when exposed to ZnPcS_4_-AuNPs induced PDT treatment (Nkune and Abrahamse, 2023).

#### 3.2.5 Live/dead assay acridine orange-ethidium bromide (AO/EB) fluorescent staining in Caco-2 monolayers and MCTSs

**FIGURE 14:**
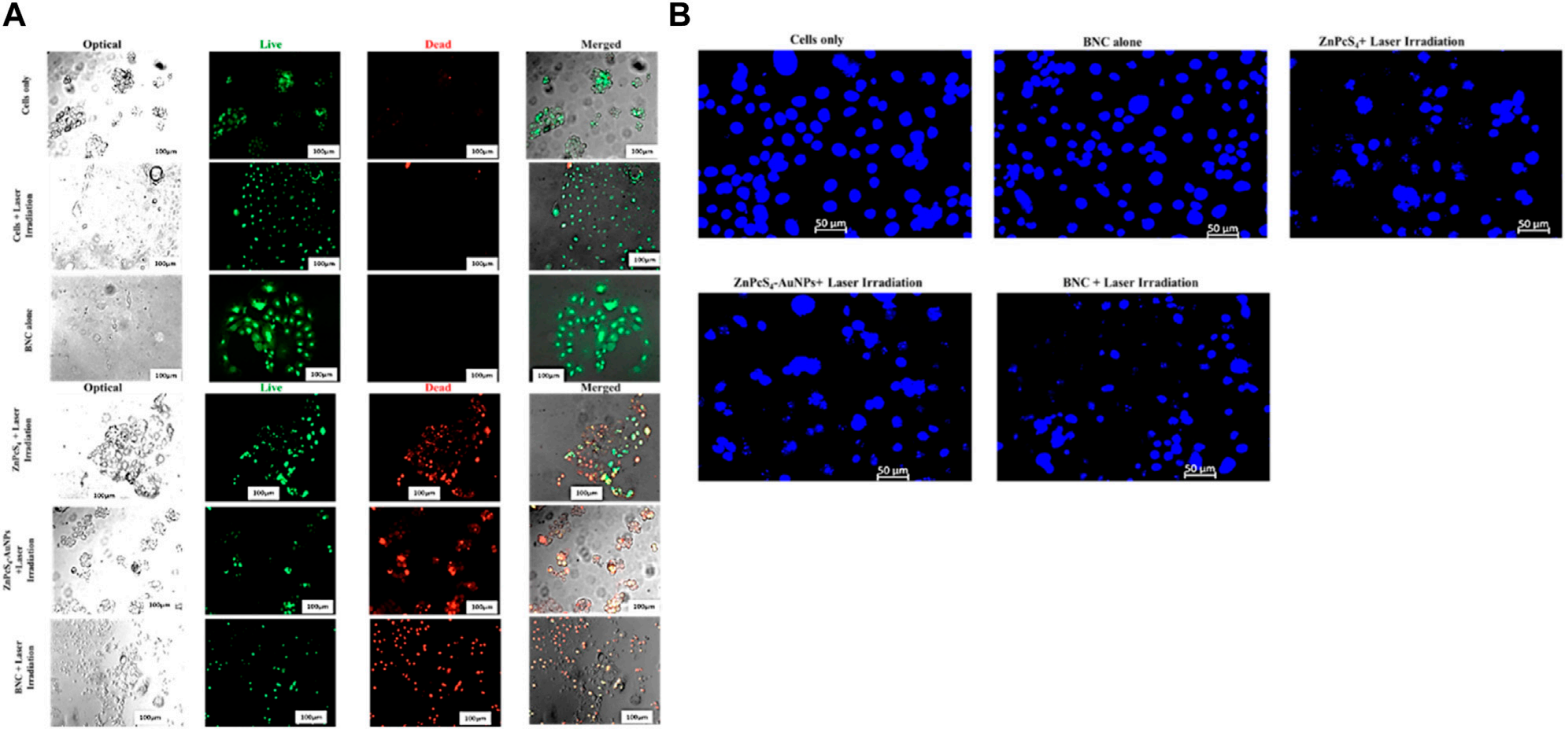
**(A)**Fluorescent microscopic images of Caco-2 monolayer cancer cell line treated with a ZnPcS_4,_ ZnPcS_4_-AuNPs or BNC and laser illumination, and stained with AO/EBr. Acridine orange highlighted green; live cells and Ethidiumbromide homodimer distinguished the dead cells in red, thereby facilitating the differentiation between live and dead cells. **(B)** Representative images of Hoechst nuclear staining in control and experimental groups. The extent of nuclear damage was pronounced in the experimental group treated with ZnPcS_4_ plus laser light, ZnPcS_4_-AuNPs plus laser light and BNC + exposure to irradiation with laser light of 673 nm at 10 J/cm^2^ treatment.

**FIGURE 15:**
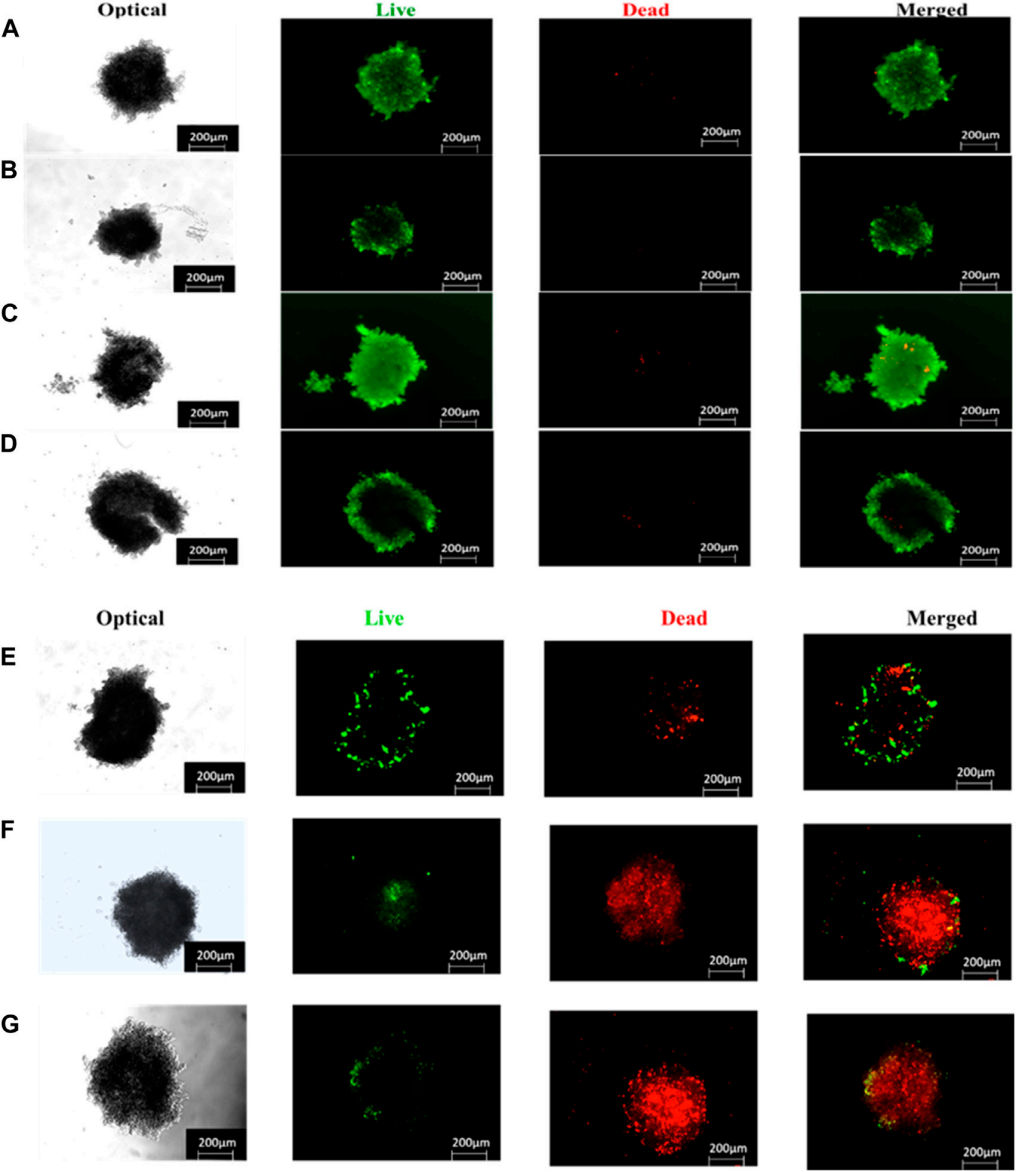
Live/dead staining (live = green and dead = red) images of ZnPcS_4,_ ZnPcS_4_-AuNPs or BNC treated Caco-2 MCTS captured with a Zeiss Live imager ×10 magnification). Scale bar = 200 µm. **(A)** Untreated MCTS, **(B)** ZnPcS_4_ PS, **(C)** ZnPcS_4_-AuNP, **(D)** BNC nanoconjugates, **(E)** ZnPcS_4_ +Irr, **(F)** ZnPcS_4_-AuNP + Irr and **(G)** BNC nanoconjugates + Irr.

Live/dead staining plays a crucial role in assessing cell viability within the scope of potential therapeutic applications involving prepared BNC nanoconjugates. We evaluated the anticancer effects of ZnPcS_4_, ZnPcS_4_-AuNPs or BNC nanoconjugate -mediated PDT using the Acridine Orange-Ethidium Bromide cell staining assay on Caco-2 monolayer cell cultures or MCTSs (Figures 14A, 15). These staining agents respectively identify live and dead cells, appearing as green and red signals. As depicted in Figures 14A, 15, the untreated control group exhibited exclusively live cells, devoid of any dead cells. In contrast, the occurrence of dead cells gradually increased in the following order: ZnPcS_4_, ZnPcS_4_-AuNPs or BNC nanoconjugates, in treated cells. While cell death was observed in all the PDT treated groups the BNC plus laser irradiated treated group, with its active targeting component, exhibited the highest ratio of dead cells to living ones, proving to be the most effective treatment approach. Therefore, the BNC plus laser irradiated treated group demonstrated a superior cell mortality rate compared to the other groups, underscoring its potential as an effective PDT treatment modality. Additionally, to evaluate the extent of DNA damage in monolayer cells, cells were stained with Hoechst stain and slided were imaged using a fluorescence microscopy. As depicted in Figure 14B. Caco-2 monolayer treated with ZnPcS_4_, ZnPcS_4_-AuNPs or BNC and exposed to irradiation with laser light of 673 nm at 10 J/cm^2^ showed nuclear shrinkage and irregular shape and fragmented nuclei, typically observed during apoptosis. In concurrence with the results of other experimental analysis, BNC-mediated PDT was responsible for an even more pronounced nuclear damage, where cells showed significant nuclear shrinkage, chromatin condensation and scattered nuclear granules of irregular shape, supporting evidence of the capability of BNC-PDT to induce higher numbers of apoptotic cells (Figure 14B).

## 4 Discussion

CRC cancer treatment options are predominantly limited to conventional techniques such as surgery, radiation, and chemotherapy; however, these approaches fall short of completely ablating this life-threatening disease (Winifred Nompumelelo Simelane and Abrahamse, 2021). This inadequacy can, to some extent, be attributed to the intrinsic characteristics of the disease, whereby genetic and epigenetic alterations within the tumours endow cancer cells with certain biological traits that not only resemble normal cells as means to evade the immune system but also confer resistance to therapeutic interventions, ultimately resulting in treatment ineffectiveness and the likehood of tumour recurrence. Furthermore, the existing therapeutic approaches are associated with numerous adverse effects, which can lead to unfavourable prognosis, and relapse (Winifred Nompumelelo Simelane and Abrahamse, 2021). Therefore, novel and efficient therapeutic strategies are constantly needed that can overcome drawbacks often encountered in classical methods (Winifred Nompumelelo Simelane and Abrahamse, 2021).

PDT is a light-based treatment which uses a photosensitizing agent that is activated by appropriate light to selectively destroy abnormal cells or tumours, while leaving surrounding healthy tissues unharmed (Kwiatkowski et al., 2018). It offers several advantages compared to some traditional treatments; including minimal invasiveness, minimal scarring and repeatable if necessary, allowing for ongoing treatment without cumulative toxicity concerns that may be present with certain conventional therapies (Correia et al., 2021). However, most traditional PSs face substantial shortcomings, including limited permeability, non-specific phototoxicity, side effects, hydrophobicity, low bioavailability, and a tendency to aggregate (Winifred Nompumelelo Simelane and Abrahamse, 2021), and such limitations pose a challenge on the effectiveness of PDT. To address these issues, actively targeting is one strategy to enhance PDT by functionalizing nanoparticles with targeting moieties, like antibodies, and/or conjugating them with photosensitizers (Simelane et al., 2021). This specific targeting approach improves PS drug permeability across biological membranes, accommodate hydrophobic compounds as well as enables the selective delivery of photosensitizers to cancer cells, thus improving PDT treatment effectiveness while minimizing collateral damage to healthy tissues (Kwiatkowski et al., 2018).

In the area of preclinical research, as well as in the evaluation of photodynamic effect associated with targeted PDT, *in vitro* 2-D cell culture models hold a prominent role and are extensively used as they are easy to prepare, maintain, and manipulate (Winifred Nompumelelo Simelane and Abrahamse, 2021). However, these models consist of basic monolayers of cells grown on flat surfaces, and thus, they are incapable of authentically reproducing the intricate microenvironment found within cells or the numerous interactions that often occur between cellular and non-cellular components (Winifred Nompumelelo Simelane and Abrahamse, 2021; Kucinska et al., 2021). Moreover, in contrast to 3-D cell cultures, 2-D monolayer cell cultures lack the appropriate microenvironment and nutritional gradient often observed within solid tumours (Kucinska et al., 2021; Dalir Abdolahinia and Han, 2023). Therefore, some of the limitations often associated with using conventional 2-D monolayer cell cultures to investigate the photocytotoxicity effects of actively targeted PDT can be addressed by adopting 3-D cell cultures such as MCTSs (Winifred Nompumelelo Simelane and Abrahamse, 2021; Rossi and Blasi, 2022). 3-D cell spheroid models are believed to promote cell-cell interaction which can potentially influence cellular growth, metabolism and cell response. Therefore, spheroids may offer a more robust and precise approach for investigating the intricate biological interactions between actively targeted NP-PS delivery systems and specific colorectal target tissues in PDT applications (Winifred Nompumelelo Simelane and Abrahamse, 2021; Rossi and Blasi, 2022; Pereira et al., 2017).

However, few studies have been conducted to investigate the NP-PS delivery systems in PDT in the *in vitro* treatment of colorectal cancer MCTSs (Winifred Nompumelelo Simelane and Abrahamse, 2021). Furthermore, as already mentioned, the absence of targeted delivery of PSs to CRC cells hampers the effectiveness of PDT (Lima and Reis, 2023). Therefore, the issue of inadequate targeting of PSs can be addressed through their encapsulation in NPs such as AuNPs, which can consequently enhance the circulation time of PSs. Moreover, functionalization of NP-PS with targeting ligand/moieties could enhance PDT efficacy, as suggested by previous studies (Zheng et al., 2020; Simelane et al., 2021). Therefore, within our study, anti GCC mAb was identified as specific and efficacious moiety for the overexpressed receptors in CRC cancer (Simelane et al., 2021). Herein, in this study, we report the used of biofunctional nanoconjugates (BNC) composed of ZnPcS_4_ bound to antibody (anti GCC-mAb) functionalized AuNPs for targeted ZnPcS_4_ PS delivery through the binding on the GCC receptors expressed on target Caco-2 cells, with the purpose of exploring the concept of active targeting, enhancing PS cellular uptake and consequently enhancing the overall PDT efficacy on human colorectal cancer cell line, Caco-2. We utilized Caco-2 monolayers and MCTSs to compare cellular responses to the biofunctional NPs post PDT.

After the synthesis and pegylation of AuNPs and the subsequent conjugation of ZnPcS_4_, the freely available terminal of the amine functionalized AuNPs with PEG 2000 of the nanoconjugates was attached to the cell targeting anti GCC. Anti GCC activation was achieved through 1-Ethyl-3-(3-dimethylaminopropyl) carbodiimide/N-hydroxy succinimide coupling reaction to form amide bonds between carboxyl groups of Anti GCC and amino groups of the nanoconjugates, to obtain the biofunctional ZnPcS_4_- GCC- loaded AuNPs (BNC) compound. Successful conjugation of pegylated AuNP with ZnPcS_4_- and anti GCC was confirmed by UV-Vis spectrophotometry analysis of the newly formed nanocompound. UV-Vis spectrophotometry analysis confirmed the presence of the absorption peaks of the individual constituents and the final BNC compound. BNC nanoconjugates exhibited three absorption peaks for ZnPcS_4_. Notably these absorption peaks in the Soret band and Q band are typically suited for photodynamic diagnosis (PDD) and photodynamic therapy (PDT) applications, respectively (Simelane et al., 2021; Olszowy et al., 2023).The BNC also presented a distinctive peak for AuNPs which was a typical absorption band found in the UV Vis spectrum of small, spherical gold nanoparticles, and an absorption peak ascribed to the anti GCC mAb was observed, which fall within the typical range of the spectra of protein in the UV Vis spectrum. These results confirmed the fabrication of the BNC and that the optical properties were retained in the nanoconjugates (Simelane et al., 2021).

The specificity of our NP-PS delivery system was achieved by functionalizing the NP-PS system with anti GCC-mAb that targets overexpressed GCC receptors in CRC cancer cells (Simelane et al., 2021; Danaee et al., 2017). The ^1^H NMR spectra analysis of the PEG-AuNP-antibody complex showed evidence of coordination and broadening observed as multiplets in the range of 3.24–3.48 ppm. Moreover, a slight shift (0.22 ppm) towards the upfield was observed, which indicated the protons on the alkoxy carbons segment.

Nanoparticle size plays a critical role in targeted photodynamic therapy, which can strongly influence their circulation, PS uptake and biodistribution (Winifred Nompumelelo Simelane and Abrahamse, 2021; Augustine et al., 2020). Studies have reported that small-sized spherical AuNPs displayed the highest antitumour potential. Moreover, the offer advantages of improved localization and internalized by cells in comparisons to larger AuNPs within PDT applications (Winifred Nompumelelo Simelane and Abrahamse, 2021; Kumar et al., 2017). Our TEM morphology assessment of BNC-nanoconjugates were observed to have spherical structures with average sizes of 13 nm, demonstrating their capability and feasibility of achieving effective delivering of PS to the diseased site. Additionally, the narrow particle size distribution of the BNC nanoparticles is likely to contribute to higher PS internalization into cancerous cells, thus can easily penetrate deeply within the tumour tissues facilitated by the enhanced permeability and retention (EPR) effect. Moreover, the decoration with anti GCC Ab on NPs could facilitate the interaction with the GCC receptors located on CRC cell membranes, thus improving PDT outcomes (Simelane et al., 2021).

In the present investigation, we determined the ATP levels post exposure to the ZnPcS_4_-loaded-Anti GCC-AuNPs nanoconjugates combined with 673 nm laser irradiation towards Caco-2 monolayers and MCTSs. As expected, ZnPcS_4_-loaded-Anti GCC-AuNPs mediated PDT induced a markedly decrease in ATP levels in both monolayers and MCTSs than in control groups, demonstrating the potential of the BNC to inhibit cell viability in both cell culture models. Moroever, this indicated favourable selectivity toward Caco-2 cancer cells, owing to the specific targeting antibody moieties-functionalized nanoparticles. However, 2D cell culture models produced a much more reduction in ATP levels than 3-D cultures. Monolayers from A375 melanoma cells have also been reported to have a markedly reduction in cell proliferation as determined by ATP assay post exposure to ZnPcS_4_ (Nkune and Abrahamse, 2023), as observed for the BNC nanoconjugates. Our findings indicated that monolayers are likely to be more susceptible to BNC- mediated PDT treatment than MCTSs, which were slightly more resistant and required a higher concentration dosage than monolayers. The slight reduction in ATP levels in 3-D cell culture models could be associated with the layered arrangement of spheroids, wherein cells in the outmost layers are subjected to nutrients and oxygen in the medium, while deeper inside the spheroid, quiescent and necrotic zones are formed, which are slightly deprived of oxygen, rendering them less susceptible to the treatment, relative to 2D structures (Winifred Nompumelelo Simelane and Abrahamse, 2021).

PDT is commonly recognized for its ability to exert selective cytotoxic effects to cancerous tissues, owing to its capability to induce the production of ROS, that can ultimately trigger cell death such as apoptosis (Gunaydin et al., 2021). Caco-2 monolayers and MCTSs were treated with BNC conjugates. The cell death mode (apoptotic cells and necrotic cells) induced by BNC mediated PDT in the Caco-2 cells cultured in 2-D and 3-D cell culture systems, were investigated through incubation of cells with Annexin V-(FITC)/PI staining, revealing that both monolayers and MCTSs were more susceptible to BNC- PDT which leads to an apoptotic cell death mode in PDT-treated cells. A distinct increase in early apoptotic and late apoptotic population in 2-D monolayers and MCTSs can be observed for the BNC treated population, confirming the effectiveness of BNC-PDT in substantially lowering cellular viability of CRC tumour cells. We also found that the rate of late apoptotic population in 3-D cell culture models was notably higher than cells maintained in 2-D culture models post PDT treatment with BNC nanoconjugates. Such an increase in late apoptotic population was accompanied by a significant decline in the live-cell population grown in 3-D culture conditions compared to the cells grown in monolayer cultures. Furthermore, a switch to necrotic populations was also observed. Our results concur with those of Nkune and others, who reported apoptosis induction after PDT which was observed via Annexin V-FITC/PI staining in A375 human melanoma cells cultured MCTSs (10 J/cm^2^) and the authors highlighted the differences in cellular responses to PDT treatment between 2-D and 3-D cell cultures (Nkune and Abrahamse, 2023).

Accumulating evidence has indicated that caspase-3 and caspase 9 play critical roles as proteases in stimulating the apoptosis pathway within zinc phthalocyanine -PDT of CRC malignant cells (Gholizadeh et al., 2021). Gholizadeh et al. have reported that ZnPc-PDT with 12 J/cm^2^ or 24 J/cm^2^ can result in upregulation of caspase-3 and caspase-9 leading to the trigger of the intrinsic apoptosis pathway (Gholizadeh et al., 2021). Consistent with these, our results have indicated that BNC-PDT increased the caspase 3 and caspase 9 activities suggesting that caspase 3 and caspase 9 most likely modulated apoptosis induced by BNC treatment, in both 2-D and 3-D cell culture systems. The effectiveness of BNC in PDT against Caco-2 monolayers and MCTSs was further confirmed through Live/Dead assay. Compared with the control groups, monolayers and MCTSs that were subjected to laser irradiation after treatment with ZnPcS_4_ exhibited pronounced red fluorescence, indicating the presence of dying cells, and a reduced green fluorescence, signifying a decrease in live cells. Notably, the group treated with BNC nanoconjugates, and exposed to laser irradiation displayed the most significant therapeutic effects. Collectively, these findings provide evidence for the remarkable antitumour potential of using ZnPcS_4_-loaded-Anti GCC-AuNPs nanoconjugates in monolayers and MCTSs. Overall, BNC mediated PDT effectively promote apoptosis, although MCTSs were less susceptible to phototoxic damage than the monolayers, thus strongly indicating the differences in 2-D and 3-D cell culture models, further emphasizing the crucial role of using 3-D cell culture models prior to *in vivo* animal experiments for targeted PDT investigations.

## 5 Conclusion

This study substantiated the potential of a multicomponent PS delivery system (ZnPcS_4_-loaded-Anti GCC-AuNPs) that was small and spherical in morphology, and displayed robust photocytotoxicity effect in PDT cancer treatment. The effectiveness of this system was evaluated using both Caco-2 2-D monolayer cell cultures and 3-D MCTSs cell culture systems. As part of this study, MCTSs were generated, which could serve as practical 3-D models for targeted PDT. The 3-D cell culture models could mimic some features of *in vivo* avascular tumour tissues, to some extent, and potentially bridge the gap between monolayer cultures and *in vivo* models. The efficacy of the nanoconjugate-PS delivery system was assessed using these MCTSs cell culture models. Notably, higher PS concentrations were required to induce cytotoxicity in Caco-2 MCTSs compared to cell monolayers, whether in the form of free PS drugs or NP-PSs nanoconjugates. This is attributed to the dense structure of MCTSs and their PS drug resistance. The nanoconjugates exhibited exceptional performance in terms of anticancer activity within PDT applications, indicating its potential success in more challenging *in vivo* animal models. Consequently, our findings underscore the significance of assessing actively targeted PDT using 3-D tumour spheroid models as an intermediary step, providing critical insights into formulating efficacy prior to conducting *in vivo* animal tests, a level of detail not attainable through conventional 2-D culture systems. The translation and exploiting of 3-D cell culture models such as MCTSs could possibly integrate other tumour microenvironment components like stromal cells and introduce a groundbreaking approach to CRC targeted PDT, potentially leading to more effective treatment.

## Data Availability

The original contributions presented in the study are included in the article/Supplementary Material, further inquiries can be directed to the corresponding author.
