# Supplementary figures and images for: Zinc phthalocyanine loaded- antibody functionalized nanoparticles enhance photodynamic therapy in monolayer (2-D) and multicellular tumour spheroid (3-D) cell cultures
Source: Front Mol Biosci. 2024 Jan 8;10:1340212. doi: 10.3389/fmolb.2023.1340212 (PMC10801020; doi:10.3389/fmolb.2023.1340212)

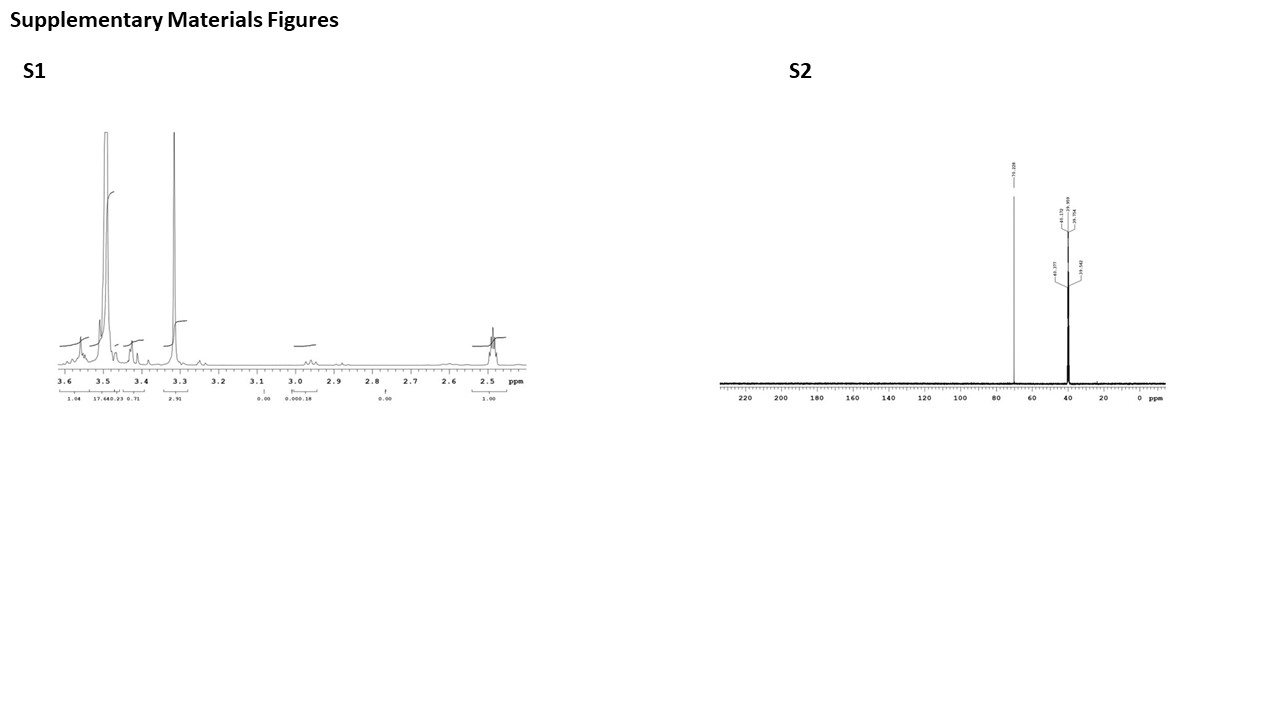

Supplement: Supplementary file 1 [file Image1.JPEG]
